# Cross-Sectional Association of Food Source with Food Insecurity, Dietary Diversity and Body Mass Index in Western Kenya

**DOI:** 10.3390/nu14010121

**Published:** 2021-12-28

**Authors:** Elizabeth Olatunji, Charles Obonyo, Pamela Wadende, Vincent Were, Rosemary Musuva, Charles Lwanga, Eleanor Turner-Moss, Matthew Pearce, Ebele R. I. Mogo, Oliver Francis, Louise Foley

**Affiliations:** 1 MRC Epidemiology Unit, University of Cambridge, Cambridge CB2 0SL, UK; eleanor.turner-moss@nhs.net (E.T.-M.); Matthew.Pearce@mrc-epid.cam.ac.uk (M.P.); ebele.mogo@mrc-epid.cam.ac.uk (E.R.I.M.); Oliver.Francis@mrc-epid.cam.ac.uk (O.F.); louise.foley@mrc-epid.cam.ac.uk (L.F.); 2 Centre for Global Health Research, Kenya Medical Research Institute, Kisumu 40100, Kenya; cobonyo65@yahoo.com (C.O.); vincentwere@gmail.com (V.W.); rmusuva.m@gmail.com (R.M.); clwanga11@gmail.com (C.L.); 3 Faculty of Education and Human Resources, Kisii University, Kisii 40200, Kenya; pamela.wadende@gmail.com

**Keywords:** food source, nutrition, malnutrition, dietary diversity, food insecurity, food security, body mass index, BMI, Kenya, supermarket, triple burden of malnutrition

## Abstract

The triple burden of malnutrition in many low- and middle-income countries (LMICs) is partly a result of changing food environments and a shift from traditional diets to high-calorie Western-style diets. Exploring the relationship between food sources and food- and nutrition-related outcomes is important to understanding how changes in food environments may affect nutrition in LMICs. This study examined associations of household food source with household food insecurity, individual dietary diversity and individual body mass index in Western Kenya. Interview-administered questionnaire and anthropometric data from 493 adults living in 376 randomly-selected households were collected in 2019. Adjusted regression analyses were used to assess the association of food source with measures of food insecurity, dietary diversity and body mass index. Notably, participants that reported rearing domesticated animals for consumption (‘own livestock’) had lower odds of moderate or severe household food insecurity (odds ratio (OR) = 0.29 (95% CI: 0.09, 0.96)) and those that reported buying food from supermarkets had lower odds of moderate or severe household food insecurity (borderline significant, OR = 0.37 (95% CI: 0.14, 1.00)), increased dietary diversity scores (Poisson coefficient = 0.17 (95% CI: 0.10, 0.24)) and higher odds of achieving minimum dietary diversity (OR = 2.84 (95% CI: 1.79, 4.49)). Our findings provide insight into the relationship between food environments, dietary patterns and nutrition in Kenya, and suggest that interventions that influence household food source may impact the malnutrition burden in this context.

## 1. Introduction

Suboptimal diet is the leading cause of death and disease worldwide [1]. A recent study found that dietary improvements could prevent one in every five deaths globally [2]. Poor diets have led to a global malnutrition crisis, wherein undernutrition, micronutrient deficiency, and overweight/obesity persist at high levels. All three forms of malnutrition can lead to poor physiological function and increase an individual’s risk of disease [3,4,5,6,7].

While most countries are experiencing some aspect of the malnutrition crisis, many low- and middle-income countries (LMICs) are facing the triple burden of malnutrition—the coexistence of undernutrition, micronutrient deficiency, and overweight/obesity [8,9]. This disproportionate burden of substantial levels of all three types of malnutrition is largely due to the effects of the nutrition transition on LMICs [10]. The nutrition transition describes the shifts in diet and activity patterns that accompany major changes in socioeconomic, demographic and food environment factors, and influence health outcomes [11]. For example, the increase in overweight and obesity in many LMICs is partly explained by a shift from traditional diets to high-calorie Western-style diets [12,13,14].

Food environment refers to the ‘physical, economic, political and socio-cultural’ conditions that influence consumers’ acquisition, preparation, and consumption of food [15,16]. While there is increasing evidence linking food environments to dietary behaviour and diet-related health outcomes, most existing studies examine this relationship in high-income countries alone [17,18]. Related to the food environment is the foodscape—‘the physical spaces and places for selling (and eating) food—the actual sites where people can find food’ [19]. The food environment changes in LMICs described by the nutrition transition [12] are accompanied by changes in how people source their food. For instance, open air markets have been the main source of purchased food in these countries, but are facing competition from large supermarkets and hypermarkets (department store combined with a supermarket) [20,21], which can provide more stable access to a variety of healthy and unhealthy foods [22].

Household food source refers to the method and/or location of food procurement and is ‘largely shaped by the existing food environment’ [15]. In turn, household food source may influence diet quantity, quality, diversity and safety, and may indirectly influence nutrition and health outcomes [15]. Food insecurity, dietary diversity, and body mass index (BMI) are three food- and nutrition-related outcomes that may be influenced by the diet and food environment changes in LMICs. Discerning the association of food source with food insecurity, dietary diversity, and BMI is important for understanding how changes in food environments might affect access to food and dietary behaviour in these countries, but there is currently a limited amount of evidence on these relationships. As such, this study aimed to assess the association of household food source with household food insecurity, individual dietary diversity, and individual BMI in Western Kenya, a LMIC experiencing the triple burden of malnutrition.

## 2. Materials and Methods

### 2.1. Country of Interest

Kenya is a lower-middle income country located in Eastern Africa [23]. The current estimated population size is about 55 million people [24]. The average life expectancy at birth is 69 years, and nearly 60% of the population is less than 25 years old [24]. Kenya is currently experiencing a shift in traditional to high-calorie Western-style diets, and a high prevalence of undernutrition in early childhood, of micronutrient deficiency in women and young children, and of overweight and obesity in adults, especially adult females [25]. In Kenya, 27% of women of reproductive age have anaemia (a sign of micronutrient deficiency). Twenty-six percent of children under 5 years are stunted. Thirty-four percent of adult females and 16% of adult males are overweight. Eleven percent of adult females and 3% of adult males are living with obesity [25]. Two counties in Western Kenya, Kisumu County and Homabay County, are the main study areas. The population of Kisumu County is 968,909 persons, and the population of Homabay County is 963,794 persons.

### 2.2. Study Design and Settings

This study used baseline data from the Hypermarket, Foodscape and Health study [21], an ongoing mixed-methods natural experimental study that aims to assess individual, household and population impacts of a new hypermarket in Western Kenya on dietary behaviours, food purchasing, physical activity patterns and BMI. In this natural experimental study, the intervention area was the Mamboleo area of Kisumu County (urban), where a new hypermarket was under construction at the time of data collection, and the control area was the Sofia area of Homabay county (peri-urban), where there is no hypermarket existing or planned. The areas were selected based on spatial census data, visits by the researchers to the study areas, and local expertise. The control area was matched to the intervention area—a 2 km radial buffer around the hypermarket—by geographical size and population density, and by similarity of socioeconomic, land terrain and food retail characteristics. Both sites have a combination of low and middle/high socioeconomic areas, major roads, a comparable number of existing supermarkets and contain a main shopping street.

Ethical approval for this study was granted by the Kenya Medical Research Institute Scientific and Ethics Review Unit (reference KEMRI/SERU/CGHR/174/3730) and the Cambridge Psychology Research Ethics Committee. All study participants received an information sheet and provided written informed consent before taking part in the study.

### 2.3. Survey Methods

Within each 2 km radius sampling frame, households were stratified by distance from the centre, socioeconomic status and sampling quadrant. From each of these strata, a random sample of households was taken. Participants were first drawn from the intervention area and then matched by demographic characteristics in the control area. At baseline, 200 households in each study area were drawn. Households headed by children aged under 18 years were excluded from the study. In addition to the household survey, up to five adults aged 18 years or over from each household were able to participate in the individual survey.

Baseline data were collected electronically in March (Kisumu) and June (Homabay) 2019 with pre-programed forms on various days of the week. The surveys were completed as interview-administered questionnaires led by trained study teams made up of two field workers and a Community Health Volunteer. The head of the household and the household member in charge of food purchasing completed the household survey. The household head was defined as the ‘adult member of the household who is accepted and recognised by the other household members as the head’ [21], and the household member in charge of food purchasing was defined as the member of the household who obtained the majority of the food for the household and was most responsible for decision making concerning household food source. The household survey included questions about household composition, food purchasing, travelling to buy food and food insecurity as well as anthropometric measurements for the children (age 17 or younger) in the household. The full household survey can be found in Supplementary Table S1. The individual survey (Supplementary Table S2) included questions about demographics, physical activity, wellbeing and social connectedness, along with anthropometry and a 24 h diet recall (Supplementary Table S3).

### 2.4. Exposures

The exposure of interest in this study was household food source, defined as the origin(s) from which food for the household was acquired during the last month (Table 1). Food source was divided into two categories: food source and food retail source. The category ‘food source’ included ‘purchased’ and several non-retail methods for food acquisition. The category ‘food retail source’ included food retail outlets from which household food was bought. The household member in charge of food purchasing reported all the food sources used to acquire food for the household. Each source was then coded as a binary yes/no variable.

### 2.5. Outcomes

#### 2.5.1. Household Food Insecurity

Responses to items in the household survey (Supplementary Table S1) were used to derive variables for household food insecurity. These are similar to items used in the Food and Agriculture Organisation’s Food Insecurity Experience Scale (FIES) [26] but with slightly altered question phrasing (see Supplementary Table S4 for details) and the addition of a question about receiving organized aid/relief food identical to that used in a recent national household survey in Kenya [27]. Survey responses were analysed using the Rasch model [26] to derive a 3-level categorical variable that described households as having ‘no food insecurity,’ ‘moderate food insecurity,’ or ‘severe food insecurity.’ This variable was then used to create the following binary categorical variables (using ‘no food insecurity’ as a reference): moderate or severe food insecurity (yes/no), and severe food insecurity (yes/no).

#### 2.5.2. Individual Dietary Diversity

Individual dietary diversity was assessed using the Minimum Dietary Diversity for Women (MDD-W) indicator from the Food and Agriculture Organisation of the United Nations [28]. The MDD-W classifies food and drinks consumed by an individual into 10 core food groups (grains, white roots, tubers and plantains; pulses; nuts and seeds; dairy; meat, poultry and fish; eggs; dark green leafy vegetables; other vitamin A–rich fruit and vegetables; other vegetables; and other fruits) and defines achieving minimum dietary diversity as having consumed greater than or equal to 5 of the 10 core food groups the previous day [28]. This dietary diversity indicator presents minimum dietary diversity ‘as a proxy to micronutrient adequacy’ for women of reproductive age [28]. Precedent for the use of MDD-W as a pragmatic approach to assess dietary diversity in adults in low resource settings can be found in a recent study [29].

Adults taking part in the individual survey completed an uninterrupted multiple-pass pen and paper 24 h diet recall. Fieldworkers coded each reported food or drink item to an appropriate food group on the electronic data collection system with guidance from a food list (Supplementary Table S5). These data were transformed into two variables: a count variable noting each individual’s dietary diversity score (range from 0–10, with a point given for each food group consumed from the 10 MDD-W core food groups), and a yes/no binary variable noting achievement of minimum dietary diversity (with minimum dietary diversity defined as a dietary diversity score of 5 or higher).

#### 2.5.3. Anthropometry

Weight and height of all participants in the individual survey were measured by trained interviewers (instruments used: 813 High-Capacity Digital Flat Scale, SECA, Hamburg, Germany; 213 Portable Stadiometer Height-Rod, SECA, Hamburg, Germany). A continuous variable for BMI (weight in kilograms divided by height in metres squared) was derived.

### 2.6. Covariates

The following self- or proxy-reported covariates were considered potential confounders of associations.

Household-related covariates included household study area (Kisumu, Homabay); household head sex (female, male); household head age (years); household head’s highest education level (up to primary, secondary, post-secondary); household head’s working status (unemployed, employed/retired); number of people living in the household (1 to 15); duration the household had resided in the local area (years and months); type of dwelling (bungalow, flat, maisonette, Swahili, Shanty, Manyatta/traditional house, other); electricity in the home (yes/no); main source of water for the household (piped water, public tap/stand pipe, well, vendors, surface water, other); fridge in the home (yes/no); access to a private car (yes/no).

Individual-related covariates included sex (female, male); age (years); self-reported physician diagnosis of hypertension (not during pregnancy), diabetes, raised blood cholesterol, heart disease, stroke or cancer (for each condition: yes, no, don’t know); self-rated overall health status (very dissatisfied, dissatisfied, neither satisfied nor dissatisfied, satisfied, or very satisfied); and physical activity (self-reported daily minutes of moderate to vigorous intensity physical activity) [30,31]. Information on the highest educational qualification, working status and household size (all coded as above) was linked to each individual from data on those variables provided for each household member in the household survey.

### 2.7. Statistics

All statistical analyses were performed using STATA (STATA 16.0; StataCorp, College Station, TX). Statistical significance for all tests was pre-determined as *p* < 0.05, and all tests were two-sided.

#### 2.7.1. Analytical Sample

A total of 23 individuals were excluded because their education and employment information could not be unambiguously linked to them from the household survey, or their individual data could not be matched with a pre-existing household ID from the household survey. Eight households with missing data for the household head were excluded from models using those covariates. Missing data were not imputed.

#### 2.7.2. Descriptive Statistics

Differences in exposure, outcomes and characteristics by study area were tested using the Wilcoxon rank sum test, Pearson’s chi-squared test, unadjusted Poisson regression and Fisher’s exact test, accounting for clustering of participants within the same household where appropriate.

#### 2.7.3. Associations between Household Food Source and Household Food Insecurity

The association of household food source with moderate or severe household food insecurity (referent: no food insecurity) and with severe household food insecurity (referent: no food insecurity) was assessed using logistic regression. The first model was unadjusted, then the regression analysis was repeated with adjustment for the following covariates: study area, household head characteristics (age, sex, highest educational qualification and working status), household size, years lived in dwelling and asset-based measures of socioeconomic status.

#### 2.7.4. Associations between Household Food Source and Individual Dietary Diversity

Poisson regression was used to assess the association of household food source with individual dietary diversity score. Logistic regression was used to assess the association of household food source with achievement of minimum individual dietary diversity (referent: not achieving). The first model was unadjusted, then the regression analysis was repeated with adjustment for the following covariates: study area, age, sex, highest educational qualification, working status, household size, self-reported non-communicable diseases, self-rated overall health and physical activity.

#### 2.7.5. Associations between Household Food Source and Individual Body Mass Index

A generalised linear model with an identity link function was used to assess the association between household food source and individual BMI. Covariates included in the adjusted model were the same as those used for the associations between household food source and individual dietary diversity. One individual’s BMI was identified as an outlier (>100 kg/m^2^) and was excluded from the BMI analyses.

#### 2.7.6. Sensitivity Analysis

A sensitivity analysis was performed in which asset-based measures of socioeconomic status were replaced with average gross monthly household income per household member in the adjusted model assessing the association between household food source and household food insecurity. The head of the household was asked to report the current average monthly household income for all employed household members. A continuous variable for average gross monthly household income per household member was then derived by dividing the reported monthly income by household size.

## 3. Results

### 3.1. Characteristics of the Study Households and Individuals

The household survey was completed by 376 (94%) out of 400 households contacted. Table 2 summarises the characteristics of these households. Statistically significant differences were observed between study areas for the following household characteristics: sex and working status of the household head, household size, type of dwelling, presence of electricity and main water source. No differences were observed between study areas for age and highest educational qualification of the household head, years living in the local area, owning a fridge or car, moderate or severe food insecurity and severe food insecurity. Eighty-four percent of households reported moderate or severe food insecurity, and 58% reported severe food insecurity. The eight households with missing data for their household heads are described in Supplementary Table S6.

A total of 493 individuals, drawn from 357 households, were included in the analyses (Table 3). The median age was 35 years, and the majority (70%) were female. The mean dietary diversity score for individuals in this sample was 4.3/10 and 44% of participants achieved minimum dietary diversity. Of the 492 individuals included, the median BMI was 23.6 kg/m^2^. Self-rated overall health and physical activity were the only characteristics for which a statistically significant difference was observed between individuals in Kisumu and Homabay. Supplementary Table S7 summarises the malnutrition characteristics for the included individuals. Overall, 10% were underweight, 43% were overweight or obese, and 56% had insufficient micronutrient adequacy (based on the Minimum Dietary Diversity for Women indicator).

Descriptive data on household food source for the included households are shown in Table 4. The most commonly reported food source was ‘purchased’ whereas the most commonly reported food retail source was ‘open air market’. Supplementary Table S8 shows descriptive data on household food source for the households from which individual participants were drawn.

### 3.2. Association between Household Food Source and Household Food Insecurity

Table 5 presents odds ratios for moderate or severe household food insecurity depending on use of different household food sources. In the adjusted model, households that reported ‘own livestock’ (OR: 0.29; 95%CI: 0.09, 0.96), as a food source or ‘specialised shop’ (OR: 0.19; 95%CI: 0.06, 0.60) or ‘restaurant’ (OR: 0.12; 95%CI: 0.03, 0.48) as food retail sources had lower odds of reporting moderate or severe household food insecurity. Households that reported ‘supermarket’ (OR: 0.37; 95%CI: 0.14, 1.00) as a food retail source also had lower odds of reporting moderate or severe household food insecurity. There was no observed association of the remaining food sources and food retail sources with moderate and severe household insecurity. The association of some sources with moderate or severe household food insecurity could not be assessed due to collinearity. For example, ‘purchased’ was a food source used by almost all households in this sample, and ‘gather/hunt/fish,’ was a food source used by very few. After adjustment for covariates, none of the household food sources or food retail sources were significantly associated with severe household food insecurity (Supplementary Table S9).

### 3.3. Association between Household Food Source and Individual Dietary Diversity

Table 6 shows Poisson regression coefficients for the association between household food source and individual dietary diversity score. In the adjusted model, a lower dietary diversity score was observed in individuals for whom either ‘purchased’ (Poisson coefficient: −0.48; 95%CI: −0.59, −0.37) or ‘relief’ (Poisson coefficient: −0.31; 95%CI: −0.60, −0.02) was reported as a household food source and in individuals for whom ‘online’ (Poisson coefficient: −0.31; 95%CI: −0.60, −0.02) was reported as a household food retail source. By contrast, individuals for whom ‘supermarket’ (Poisson coefficient: 0.17; 95%CI: 0.10, 0.24) was reported as a household food retail source reported higher dietary diversity scores.

Supplementary Table S10 displays the results of the regression analysis examining the association between household food source and achievement of minimum dietary diversity. After controlling for covariates, individuals for whom ‘supermarket’ (OR: 2.84; 95%CI: 1.79, 4.49) or ‘kiosk’ (OR: 1.67; 95%CI: 1.02, 2.74) was reported as a household food retail source had increased odds of achieving minimum dietary diversity.

### 3.4. Association between Household Food Source and Individual BMI

The association between household food source and individual BMI is presented in Table 7. Following adjustment for covariates, individuals for whom ‘purchased’ was reported as a household food source had a lower BMI (in kg/m^2^) than individuals who did not use this source (linear coefficient: −3.25; 95%CI: −4.89, −1.62). A higher BMI was observed in individuals who had ‘gather/hunt/fish’ reported as a household food source (linear coefficient: 3.66; 95%CI: 0.52, 6.80). Lastly, individuals who had ‘online’ reported as a household food retail source had a lower BMI (linear coefficient: −4.17; 95%CI: −6.16, −2.18).

### 3.5. Sensitivity Analysis

A sensitivity analysis replacing asset-based measures of socioeconomic status with average gross monthly household income found similar results with the original analysis with a few exceptions (Supplementary Table S11). Use of average gross monthly household income per household member as a covariate saw the strengthening of the significant association between food retail source ‘supermarket’ and moderate or severe household food insecurity, and the emergence of an association between food retail source ‘supermarket’ and severe household food insecurity (OR:0.47; 95%CI: 0.27, 0.83). It also resulted in the dissipation of the association between food retail source ‘restaurant’ and moderate or severe household food insecurity (OR:0.27; 95%CI: 0.06, 1.20).

## 4. Discussion

This study explored the association of household food source (incorporating retail and non-retail sources, and different types of food retail outlets) with household food insecurity, individual dietary diversity, and individual body mass index in Western Kenya. Our findings indicated that household food source was associated with these outcomes, comparing households that used a particular food source with those that did not. Notably, reporting rearing domesticated animals for consumption (food source ‘own livestock’) was associated with lower odds of moderate or severe food insecurity and reporting buying food from supermarkets (food retail source ‘supermarket’) was associated with lower odds of moderate or severe household food insecurity, increased dietary diversity score and higher odds of achieving minimum dietary diversity. To our knowledge, this is the first study to examine the association of food source with food insecurity following adjustment for relevant covariates. Additionally, it is the first to examine the association of a wide variety of non-retail food sources with the outcomes of interest. The significance of this exploration also comes from its LMIC setting, as the impact of changes in the food environment in LMICs, including the triple burden of malnutrition, is a growing concern.

We observed several significant associations, but it is important to distinguish those that may be spurious from those that are more reliable. For example, the observed associations of food sources ‘purchased,’ ‘relief,’ ‘gather/hunt/fish,’ and food retail sources ‘restaurant’ and ‘online,’ with the outcomes of interest may be dubious due to the distribution of the data. There were very few participants in this study sample that either used or did not use these sources, and each regression model directly compared households—or individuals in households—that used a specific food source to those that did not. The associations of food source ‘own livestock,’ and food retail sources ‘supermarket,’ ‘kiosk,’ and ‘specialised shop’ with the outcomes of interest may be more reliable, as there was greater heterogeneity in the utilisation of these sources in this sample. Nine percent of households reported ‘own livestock’ as a food source, 51% reported ‘supermarket’ as a food retail source, 76% reported ‘kiosk’ as a food retail source, and 12% reported ‘specialised shop’ as a food retail source. Accordingly, these latter associations will be the focus of the remainder of this discussion. Households that sourced their food through their own livestock, from specialised shops, or from supermarkets had lower odds of moderate or severe household food insecurity than households that did not use these sources. Individuals in households that sourced their food from supermarkets had a higher individual dietary diversity score than individuals in households that did not use this source. Individuals in households that sourced their food from supermarkets or kiosks had higher odds of achieving minimum dietary diversity than individuals in households that did not utilize these sources. When the food sources with limited heterogeneity of use are disregarded, there are no associations of the food sources investigated with individual body mass index.

One previous study was identified that investigated the association between food source and food security. Because this previous study investigated individual food security rather than household food insecurity and did not adjust for potential confounders of the association, it may not be directly comparable to our current study; nonetheless, the previous study identified an association between food source and food security [32]. Our observation of an association of food source with dietary diversity is consistent with other existing studies [29,33,34,35,36]. However, the specific food sources investigated were not always commensurate with those in this study, likely due to country differences, food outlet classification and the prevalence of use of different food sources in different study samples. One study in Central Kenya found no association between shopping at a supermarket and dietary diversity score [36]. This was in direct contrast to this study, which found a positive and significant effect of reporting ‘supermarket’ as a household food retail source with dietary diversity score and achievement of minimum dietary diversity. However, the previous study examined dietary diversity at the household level, while this study examined dietary diversity at the individual level. Haynes et al. found no association of using a supermarket/wholesaler as a food source more than weekly with dietary diversity score in Fiji and Saint Vincent and the Grenadines [29]. These differences can partly be explained by considerations of the frequency of supermarket/wholesaler shopping in the Haynes et al. study compared to whether food shopping was done at a supermarket or not.

Several studies have reported an association of food source with BMI [34,35,37,38,39,40]. As noted previously, the inferential analyses concerning BMI in this study revealed an association of food source with individual BMI. However, these results were regarded as possibly spurious due to the lack of heterogeneity in the use of food sources for which a significant association with BMI was found (i.e., ‘purchased,’ ‘gather/hunt/fish,’ and ‘online’). Consequently, the association of food source with BMI seen in previous studies does not align with the interpretation of the results of this study. This difference may be explained by the right skew of the BMI distribution in this study sample.

While malnutrition is a growing problem around the world, the triple burden of malnutrition is largely unique to LMICs, where the phenomenon is threatened by food insecurity, insufficient dietary diversity, and rising BMIs. We observed a high prevalence of underweight (10%), overweight/obesity (43%), and micronutrient inadequacy (56%) in the adults in our sample. This was expected based on Kenyan national statistics on underweight and overweight/obesity [41] and the Kenya National Food and Nutrition Security Policy, which noted that most Kenyans live on diets that lack nutritional diversity [42]. The malnutrition burden in this sample highlights the importance of augmenting our understanding of the relationships between changing food environments and food- and nutrition-related outcomes in LMICs. Our findings indicate that household food source is associated with household food insecurity and individual dietary diversity, suggesting that public health interventions that influence household food source may impact food- and nutrition-related outcomes, and have downstream influences on malnutrition.

The observed association of shopping at a supermarket with household food insecurity (incorporating the original analysis and the sensitivity analysis) and with individual dietary diversity was notable. Households that reported shopping for food at supermarkets had lower odds of food insecurity and household members with more diverse diets than households that did not report using supermarkets. These findings suggest that the increasing presence and utilisation of supermarkets in LMICs may have positive consequences for food insecurity and dietary diversity across the population. Assuming these associations were true and causal, existing literature may explain these results—supermarkets often sell a wide variety of food items at lower prices than smaller independent shops. This shows that for the same amount of money, a household can buy more diverse food items at a supermarket compared with a smaller shop [43,44,45]. Although we found supermarket food purchasing to have positive implications for household food insecurity and individual dietary diversity, it is important to consider the negative consequences that supermarkets may have on processed food consumption and the prevalence of overweight/obesity [46,47].

Pre-existing interventions targeting food security, dietary diversity and malnutrition in LMICs aimed to improve household and individual buying power (e.g., cash transfers), indirectly reduce the cost of food (e.g., food vouchers and subsidies), or increase social support (e.g., community grants and village savings and loans) [48]. Empirical evidence has found that interventions that augment buying power or indirectly reduce food cost may improve food security and dietary diversity, although their effect on adult BMI is still unclear [48]. The high proportion of households in this study that reported purchasing food suggests that interventions such as these, that make purchased food more affordable, could be beneficial. The most commonly used household food retail source in this sample was ‘open air market’ (used by 99% of households). This highlights the continued reliance on open air markets in this sample despite the rise of supermarkets, suggesting that interventions aimed at reducing food costs at open air markets could have far-reaching impacts on food security and dietary diversity in this context. Besides purchasing, a fair proportion of households also procured their food through other methods, such as by rearing their own animals for consumption (‘own livestock’). Future studies are still necessary to corroborate the associations observed in this study. However, assuming the inverse association of ‘own livestock’ with moderate or severe food insecurity was true and causal, it implies that more interventions targeting food insecurity could expand beyond purchased food to incorporate other food sources that are associated with better food insecurity outcomes, such as ‘own livestock.’ For example, an initiative providing households or communities with the resources to sustainability rear their own animals for consumption. However, such an intervention may have limited value in urban areas due to complications surrounding land pressures, hygiene, disease spread and environmental pollution [49,50]. Related to this are the existing interventions that give households or communities the resources to grow their own food in local gardens [51,52]. In this study, no association was found between growing food and household food insecurity, individual dietary diversity, or individual BMI. This suggests that such interventions may not be effective in this setting and highlights the importance of public health interventions that are contextually relevant to the lived experiences of the population(s) of interest.

Strengths of our study include its examination of food- and nutrition-related exposures and outcomes at the household and individual level, as opposed to an ecological level, its evaluation of both retail and non-retail food sources with the outcomes of interest, the high response rate (94%) of contacted randomly selected households, the low number of missing data due to utilization of interview-administered questionnaires, and the setting in a LMIC. There are limitations of our study that are important for us to note. The first relates to the nature and size of our study. Due to the cross-sectional nature of this study, we could not establish a temporal relationship between our exposure and outcomes. Due to our modest sample size, we could not conduct subgroup analyses, including the assessment of effect modification by relevant covariates. Next, many of the variables were based on self-report, which may be subject to recall bias and misspecification. Household food source was used as the exposure even when the outcome was for individual-level data. While this was the most reasonable way to collect data on food source at the time (since not every individual in a household may take part in acquiring food), it disregarded intra-household food use and distribution. Lastly, the Minimum Dietary Diversity for Women indicator was used to assess dietary diversity. This indicator was validated as an appropriate tool for assessing micronutrient adequacy in women of reproductive age, but in this study, it was used to determine dietary diversity scores and achievement of minimum dietary diversity for adults aged 18 and over, regardless of their sex. While there is a precedent for use of the MDD-W to examine dietary diversity in adults in low resource settings [29], utilisation of a dietary diversity tool validated for use in all adults aged 18 and over may have been more indisputable.

Follow-up data to be collected on the sample in this study will allow for longitudinal observations of the association between the exposure and the outcomes of interest [21]. Future studies should investigate intra-household variations and the associations of food source with BMI and dietary diversity in both adults and children, to get a better understanding of how age group may modify these associations. Such investigations could also provide beneficial contributions to intervention planning. For example, a food source-related intervention that decreases stunting in children but increases BMI in adults may not be an efficacious or overall beneficial use of resources. Future work should also evaluate additional factors outside of age that may modify the association of food source with food insecurity, dietary diversity, or BMI. A particularly interesting factor to consider for food retail sources would be the method of travel to purchase food (i.e., walk, cycle, car). Lastly, additional studies should consider policies or interventions that could foster a positive relationship between supermarket shopping and healthy eating in LMICs—especially considering their increasing presence in these countries. These further investigations, carried out in Western Kenya, other areas of Kenya, and/or in other LMICs, would be beneficial to augmenting our understanding of the relationship between changing food environments and food- and nutrition-related outcomes in LMICs.

## 5. Conclusions

This study provided evidence that household food source is associated with household food insecurity and individual dietary diversity, including favourable associations of food source ‘own livestock’ with food insecurity (OR = 0.29 (95% CI: 0.09, 0.96)) and of food retail source ‘supermarket’ with food insecurity (borderline significant, OR = 0.37 (95% CI: 0.14, 1.00)) and dietary diversity (dietary diversity score—Poisson coefficient = 0.17 (95% CI: 0.10, 0.24); achieving minimum dietary diversity—OR = 2.84 (95% CI: 1.79, 4.49)). These results suggest that modifying household food source may be a valuable tool for public health interventions that aim to target food insecurity and dietary diversity, and therefore have important implications for LMICs affected by the triple burden of malnutrition while undergoing changes to the food environment as part of the nutrition transition. Follow-up data on this sample and future research including effect modification explorations of the associations of interest, will help to provide more insight into the impact of changing food environments on dietary patterns and nutrition in LMICs.

## Figures and Tables

**Table 1:** Descriptions of food sources and food retail sources included in the household survey.

**Food Source**	**Description**
Purchased (yes/no)	Buying food from a food retail outlet
Own produce (yes/no)	Farm-produced crops, such as fruits and vegetables, grown by the household
Own livestock (yes/no)	Domesticated animals raised by the household for consumption
Gift (yes/no)	Food given to the household without expectation of repayment
Relief (yes/no)	Food given to the household for the purpose of social welfare
Payment in kind (yes/no)	Food exchanged between neighbours
Gathering/hunting/fishing (yes/no)	Food acquired by foraging, hunting or fishing
Other	Any other food source
**Food Retail Source**	**Description**
Supermarket (yes/no)	A large store selling a variety of food and household items
Open air market (yes/no)	A public market where food and merchandise are sold by local vendors
Kiosk (yes/no)	A very small open-front structure that sells a small selection of food and other goods
General shop (yes/no)	A small, local shop that sells commonly used goods
Specialised shop (yes/no)	A small store that sells a specific category of goods
Informal (roadside) vendor (yes/no)	Street vendors without a permanent, fixed structure
Restaurant (yes/no)	A place where people eat prepared meals
Fast food (yes/no)	A place where people eat quickly prepared processed meals
Café (yes/no)	A place where people can order hot and cold beverages, and eat light meals
Online (yes/no)	A website that sells unprepared food items over the Internet and can have them delivered to people’s homes/communities
Other	Any other food retail outlet

**Table 2:** Characteristics of households included in analyses.

	Overall	Kisumu	Homabay	*p*-Value **
Study area (*n* = 376), *n*	376	180	196	
Female household head (*n* = 369)	174 (47)	103 (57)	71 (38)	<0.001 *
Age of household head (years, *n* = 368), median (IQR)	40 (23)	40 (21)	41 (24)	0.6
Highest educational qualification of household head (*n* = 369)				0.14
Primary or less	201 (55)	104 (58)	97 (52)	
Secondary	93 (25)	47 (26)	46 (24)	
Post-secondary	75 (20)	29 (16)	46 (24)	
Working status of household head (*n* = 369)				0.032 *
Unemployed	116 (31)	47 (26)	69 (37)	
Employed/retired	253 (69)	133 (74)	120 (63)	
Household size (*n* = 376), median (IQR)	4 (3)	3 (3)	4 (3)	0.0044 *
Years living in the local area (*n* = 376), median/IQR	12.9 (23)	14.8 (25)	12 (20)	0.78
Type of dwelling (*n* = 376)				<0.001 *
Bungalow	49 (13)	23 (13)	26 (13)	
Flat	40 (11)	7 (4)	33 (17)	
Maisonette	18 (5)	12 (7)	6 (3)	
Swahili	21 (6)	11 (6)	10 (5)	
Shanty	155 (41)	94 (52)	61 (31)	
Manyatta/traditional house	55 (15)	13 (7)	42 (22)	
Other	38 (10)	20 (11)	18 (9)	
Electricity in household (*n* = 376)	241 (64)	135 (75)	106 (54)	<0.001 *
Main water source (*n* = 376)				<0.001 *
Piped water	90 (24)	41 (23)	49 (25)	
Public tap/standpipe	157 (42)	115 (64)	42 (21)	
Well	27 (7)	11 (6)	16 (8)	
Vendors	33 (9)	10 (5)	23 (12)	
Surface water	61 (16)	2 (1)	59 (30)	
Other	8 (2)	1 (1)	7 (4)	
Own fridge (*n* = 376)	58 (15)	27 (15)	31 (16)	0.83
Own car (*n* = 376)	36 (10)	16 (9)	20 (10)	0.67
Moderate or severe food insecurity (*n* = 376)	317 (84)	148 (82)	169 (86)	0.286
Severe food insecurity (*n* = 376)	219 (58)	100 (56)	119 (61)	0.311

Note: results presented as *n* (%) unless otherwise indicated. IQR = interquartile range. * Significant at the 0.05 level. ** Pearson’s chi-squared, Fisher’s exact test, or the Wilcoxon rank sum test was used to determine statistical differences between study areas.

**Table 3:** Characteristics of individuals included in analyses.

	Overall	Kisumu	Homabay	*p*-Value **
Total individuals	493	256	237	
Total households/clusters	357	176	181	
Age (years, *n* = 493), median (IQR)	35 (22)	36 (22)	34 (23)	0.73
Female (*n* = 493)	347 (70)	183 (71)	164 (69)	0.58
Education (*n* = 493)				0.19
Primary or less	276 (56)	141 (55)	135 (57)	
Secondary	132 (27)	77 (30)	55 (23)	
Post-secondary	85 (17)	38 (15)	47 (20)	
Working status (*n* = 493)				0.17
Unemployed	223 (45)	108 (42)	115 (49)	
Employed/retired	270 (55)	148 (58)	122 (52)	
Household size (*n* = 493), median (IQR)	4 (3)	4 (3)	4 (3)	0.19
Ever had hypertension? (*n* = 493)				0.63
Yes	70 (14)	40 (16)	30 (13)	
No	418 (85)	213 (83)	205 (87)	
Don’t know	5 (1)	3 (1)	2 (1)	
Ever had diabetes? (*n* = 493)				0.85
Yes	8 (2)	5 (2)	3 (1)	
No	481 (97)	249 (97)	232 (98)	
Don’t know	4 (1)	2 (1)	2 (1)	
Ever had raised blood cholesterol? (*n* = 493)				0.5
Yes	3 (0)	0	3 (1)	
No	477 (97)	249 (97)	228 (96)	
Don’t know	13 (3)	7 (3)	6 (3)	
Ever had heart disease? (*n* = 493)				0.62
Yes	13 (3)	7 (3)	6 (3)	
No	476 (96)	248 (97)	228 (96)	
Don’t know	4 (1)	1 (0)	3 (1)	
Ever had a stroke? (*n* = 493)				0.63
Yes	11 (2)	6 (2)	5 (2)	
No	480 (98)	248 (97)	232 (98)	
Don’t know	2 (0)	2 (1)	0	
Ever had cancer? (*n* = 493)				0.63
Yes	2 (0)	0	2 (1)	
No	487 (99)	254 (99)	233 (98)	
Don’t know	4 (1)	2 (1)	2 (1)	
Self-rated overall health status (*n* = 493)				0.0073 *
Very dissatisfied	16 (3)	10 (4)	6 (3)	
Dissatisfied	139 (28)	54 (21)	85 (36)	
Neither satisfied nor dissatisfied	71 (14)	44 (17)	27 (11)	
Satisfied	229 (47)	132 (52)	97 (41)	
Very satisfied	38 (8)	16 (6)	22 (9)	
Minutes of MVPA per day (*n* = 493), median (IQR)	180 (351)	110 (334)	240 (334)	<0.001 *
Dietary diversity score (*n* = 493, 357 clusters), mean (SD)	4.3 (1.5)	4.5 (1.6)	4.2 (1.5)	0.129
Achieved dietary diversity (*n* = 493, 357 clusters)	219 (44)	125 (49)	94 (40)	0.07
Dietary recall for weekend day (*n* = 493, 357 clusters)	147 (30)	71 (28)	76 (32)	0.41
BMI (kg/m^2^, *n* = 492, 356 clusters), median (IQR)	23.6 (7.7)	23.6 (7.4)	23.5 (8.0)	0.84

Note: results presented as *n* (%) unless otherwise indicated. IQR = interquartile range. MVPA = moderate to vigorous intensity physical activity * Significant at the 0.05 level. ** Pearson’s chi-squared, unadjusted Poisson regression, or the Wilcoxon rank sum test was used to determine statistical differences between study areas.

**Table 4:** Number and proportion of 376 included households reporting use of different food sources.

	Overall	Kisumu	Homabay	*p*-Value **
	*n* (%)	*n* (%)	*n* (%)	
Food source				
Purchased	374 (99)	180 (100)	194 (99)	0.5
Own produce	133 (35)	35 (19)	98 (50)	<0.001 *
Own livestock	34 (9)	14 (8)	20 (10)	0.41
Gift	17 (5)	12 (7)	5 (3)	0.08
Relief	4 (1)	1 (1)	3 (2)	0.62
Payment in kind	2 (1)	0	2 (1)	0.5
Gather/hunt/fish	5 (1)	0	5 (3)	0.062
Barter	0	0	0	-
Other	0	0	0	-
Food retail source				
Supermarket	190 (51)	89 (49)	101 (52)	0.69
Open air market	357 (95)	165 (92)	192 (98)	0.008 *
Kiosk	286 (76)	147 (82)	139 (71)	0.015 *
General shop	151 (40)	90 (50)	61 (31)	<0.001 *
Specialised shop	46 (12)	44 (24)	2 (1)	<0.001 *
Informal (roadside) vendor	78 (21)	46 (26)	32 (16)	0.027 *
Restaurant	16 (4)	8 (4)	8 (4)	0.86
Fast food	9 (2)	4 (2)	5 (3)	1
Café	1 (0)	0	1 (1)	1
Online	1 (0)	1 (1)	0	0.48
Other	0	0	0	-

* Significant at the 0.05 level. ** Pearson’s chi-squared test or Fisher’s exact test was used to determine statistical differences between study areas.

**Table 5:** Associations of household food source and food retail source with moderate or severe household food insecurity.

	Outcome: Moderate or Severe Household Food Insecurity	
	Crude Odds Ratio (95% Confidence Interval)	Adjusted Odds Ratio (95% Confidence Interval)
**Food source (yes/no)**		
Own produce (374/2)	1.30 (0.71, 2.36)	1.26 (0.44, 3.57)
Own livestock (133/243)	0.29 (0.14, 0.63) *	0.29 (0.09, 0.96) *
Gift (17/359)	1.42 (0.32, 6.36)	0.77 (0.12, 5.07)
Payment in kind (2/374)	0.18 (0.01, 2.98)	0.20 (0.00, 53.47)
**Food retail source (yes/no)**		
Supermarket (190/186)	0.14 (0.07, 0.30) *	0.37 (0.14, 1.00) *
Open air market (357/19)	0.62 (0.14, 2.75)	0.75 (0.12, 4.73)
Kiosk (286/90)	1.22 (0.65, 2.29)	1.05 (0.43, 2.55)
General shop (151/225)	0.76 (0.44, 1.33)	1.01 (0.44, 2.30)
Specialised shop (46/330)	0.20 (0.10, 0.38) *	0.19 (0.06, 0.60) *
Informal (roadside) vendor (78/298)	1.55 (0.72, 3.30)	0.46 (0.17, 1.27)
Restaurant (16/360)	0.17 (0.06, 0.46) *	0.12 (0.03, 0.48) *
Fast food (9/367)	0.14 (0.04, 0.53) *	0.19 (0.03, 1.27)

Note: “Purchased”, “Gather/hunt/fish”, “Barter”, “Other” food sources and “Café”, “Online”, and “Other” food retail sources were omitted due to collinearity. Odds of moderate or severe household food insecurity in those using the food source vs. not using the food source. * Significant at the 0.05 level. Crude model: unadjusted, *n* = 376. Adjusted model: adjusted for study area, household head characteristics (age, sex, highest educational qualification, and working status of household head), household size and years lived in dwelling, asset-based measures of socioeconomic status (type of dwelling, electricity, main water source, owning a fridge, owning/having access to a private car), *n* = 368 (8 households missing data for household head).

**Table 6:** Associations of household food source and food retail source with individual dietary diversity score.

	Outcome: Individual Dietary Diversity Score (0–10)	
	Crude Poisson Coefficient (95% Confidence Interval)	Adjusted Poisson Coefficient (95% Confidence Interval)
**Food source (yes/no)**		
Purchased (356/1)	−0.48 (−0.52, −0.45) *	−0.48 (−0.59, −0.37) *
Own produce (124/233)	−0.01 (−0.09, 0.07)	0.04 (−0.03, 0.12)
Own livestock (33/324)	0.06 (−0.06, 0.17)	0.07 (−0.03, 0.17)
Gift (17/340)	0.10 (−0.06, 0.26)	0.07 (−0.09, 0.24)
Relief (4/353)	−0.45 (−0.75, −0.16) *	−0.31 (−0.60, −0.02) *
Payment in kind (2/355)	0.15 (−0.41, 0.70)	0.04 (−0.49, 0.57)
Gather/hunt/fish (5/352)	−0.03 (−0.39, 0.33)	0.01 (−0.34, 0.37)
**Food retail source (yes/no)**		
Supermarket (180/177)	0.20 (0.13, 0.27) *	0.17 (0.10, 0.24) *
Open air market (339/18)	−0.14 (−0.27, −0.002) *	−0.10 (−0.23, 0.02)
Kiosk (275/82)	0.06 (−0.02, 0.15)	0.07 (−0.01, 0.15)
General shop (144/213)	0.005 (−0.07, 0.08)	−0.02 (−0.10, 0.05)
Specialised shop (46/311)	0.11 (0.02, 0.21) *	0.06 (−0.05, 0.17)
Informal (roadside) vendor (74/283)	0.06 (−0.02, 0.14)	0.05 (−0.03, 0.13)
Restaurant (16/341)	0.16 (0.03, 0.28) *	0.10 (−0.03, 0.23)
Fast food (9/348)	0.04 (−0.17, 0.26)	0.02 (−0.14, 0.17)
Café (1/356)	0.15 (0.11, 0.18) *	0.27 (−0.11, 0.65)
Online (1/356)	−0.21 (−0.25, −0.17) *	−0.28 (−0.43, −0.13) *

Note: “Barter”, “Other” food sources and “Other” food retail sources were omitted due to collinearity. Poisson coefficient is the difference in log count of individual dietary diversity score for those using the food source vs. not using the food source. * Significant at the 0.05 level. Crude model: unadjusted, *n* = 493 (357 clusters). Adjusted model: adjusted for study area, age and sex, highest educational qualification, working status and household size, non-communicable diseases (hypertension, diabetes, raised blood cholesterol, heart disease, stroke, cancer), self-rated overall health and physical activity, *n* = 493 (357 clusters).

**Table 7:** Associations between household food source and food retail source with individual body mass index.

	Outcome: Individual Body Mass Index (kg/m^2^)	
	Crude Linear Coefficient (95% Confidence Interval)	Adjusted Linear Coefficient (95% Confidence Interval)
**Food source (yes/no)**		
Purchased (355/1)	−4.28 (−4.98, −3.58) *	−3.25 (−4.89, −1.62) *
Own produce (124/232)	−0.85 (−2.15, 0.45)	−0.92 (−2.30, 0.45)
Own livestock (33/323)	0.77 (−0.90, 2.43)	0.22 (−1.20, 1.63)
Gift (16/340)	0.61 (−1.88, 3.10)	0.53 (−1.79, 2.86)
Relief (4/352)	0.13 (−6.26, 6.51)	0.29 (−4.68, 5.26)
Payment in kind (2/354)	4.98 (4.28, 5.69) *	2.83 (−1.48, 7.14)
Gather/hunt/fish (5/351)	2.56 (−0.05, 5.17)	3.66 (0.52, 6.80) *
**Food retail source (yes/no)**		
Supermarket (180/176)	1.31 (−0.06, 2.68)	0.79 (−0.32, 1.90)
Open air market (338/18)	0.04 (−2.16, 2.24)	−0.43 (−2.42, 1.55)
Kiosk (274/82)	−1.68 (−3.91, 0.54)	−1.61 (−3.55, 0.33)
General shop (144/212)	0.77 (−0.74, 2.29)	0.97 (−0.66, 2.60)
Specialised shop (46/310)	0.29 (−1.61, 2.20)	0.61 (−1.29, 2.51)
Informal (roadside) vendor (74/282)	0.22 (−1.21, 1.66)	0.13 (−1.15, 1.41)
Restaurant (16/340)	5.98 (−1.02, 12.99)	5.94 (−0.80, 12.68)
Fast food (9/347)	−1.25 (−7.07, 4.57)	−2.21 (−7.72, 3.31)
Café (1/355)	9.75 (9.05, 10.45) *	1.35 (−6.51, 9.22)
Online (1/355)	−4.89 (−5.59, −4.19) *	−4.17 (−6.16, −2.18) *

Note: “Barter”, “Other” food sources and “Other” food retail sources were omitted due to collinearity. Linear coefficient is the difference in body mass index (kg/m^2^) for those using the food source vs. not using the food source. * Significant at the 0.05 level. Crude model: unadjusted, *n* = 492 (356 clusters). Adjusted model: adjusted for study area, age and sex, highest educational qualification, working status and household size, non-communicable diseases (hypertension, diabetes, raised blood cholesterol, heart disease, stroke, cancer), self-rated overall health and physical activity, *n* = 492 (356 clusters).

## Data Availability

Study meta-data are available on request. Non-identifiable individual-level data are available on request. Requests for data sharing will be considered by the principal investigators (C.O. and L.F.) in consultation with the other investigators. Their consent prevents the authors from making these data available publicly; third-party researchers would need to sign a collaborative agreement. The authors’ data sharing policies and processes meet the requirements and expectations of MRC policy on sharing of data from population and patient cohorts: http://www.mrc.ac.uk/research/research-policy-ethics/data-sharing/policy/ (accessed 19 November 2021). These policies and processes are in place to ensure that the use of data from this study is within the bounds of consent given previously by study members, complies with MRC guidance on ethics and research governance, and meets rigorous MRC data security standards.

## Supplementary Materials

The following are available online at https://www.mdpi.com/article/10.3390/nu14010121/s1, Table S1. Household survey, Table S2. Individual survey, Table S3. Diet recall sheet used alongside individual survey, Table S4. Questions from the household survey used to ascertain household food insecurity, Table S5. Food list used by fieldworkers to classify consumed items from the diet recall into appropriate food groups, Table S6. Characteristics of households with and without missing data on household head, Table S7. Malnutrition characteristics for individuals included in analyses, Table S8. Number and proportion of 357 households (from which individual participants were drawn) reporting use of different food sources, Table S9. Association between household food source and food retail source with severe household food insecurity, Table S10. Association of household food source and food retail source with achieving minimum dietary diversity, Table S11. Association of household food source and food retail source with household food insecurity.
