# Supplementary material for: Cross-Sectional Association of Food Source with Food Insecurity, Dietary Diversity and Body Mass Index in Western Kenya
Source: Nutrients. 2021 Dec 28;14(1):121. doi: 10.3390/nu14010121 (PMC8747304; doi:10.3390/nu14010121)
Supplement: Supplementary file 1 [file nutrients-14-00121-s001.zip › nutrients-1498676-supplementary.pdf]

SUPPLEMENTARY TABLES

Supplementary Table S1 Household survey.

#	Question	Answer
**Section 1: Interview details** [Interviewer to complete]		
1.1	Household ID	
1.2	Name of household head	
1.3	Name of person usually responsible for food purchasing	
1.4	Address	
1.5	GPS coordinate of dwelling: Latitude	
1.6	GPS coordinate of dwelling: Longitude	
1.7	Interviewer ID	
1.8	Completion	Interview completed<br>Interview partly completed
1.9	Visit 1 date	dd/mm/yyyy
1.10	Visit 1 time started	hh:mm
1.11	Visit 1 time ended	hh:mm
1.12	Visit 2 date	dd/mm/yyyy
1.13	Visit 2 time started	hh:mm
1.14	Visit 2 time ended	hh:mm
1.15	Visit 3 date	dd/mm/yyyy
1.16	Visit 3 time started	hh:mm
1.17	Visit 3 time ended	hh:mm
1.18	Main language of interview	English<br>Kiswahili<br>Luo
1.19	Supervisor signature	
1.20	Supervisor date	dd/mm/yyyy
1.21	Data entry signature	
1.22	Data entry date	dd/mm/yyyy
**Section 2: Household composition** [Interviewer to complete with head of household]		
2.1	How long has the household resided in the local area?	[years and months]
2.2	Please give the names of all people currently living in this household?	[List up to 15 individuals]

2.3	For each member of the household:<br>How old is [name]?	 xx years (for children under 5 years, collect age in years and months)
2.4	What is [name]'s gender?	Male<br>Female
2.5	How is [name] related to the household head?	Head<br>Spouse<br>Co-wife<br>Son/daughter<br>Spouse of son/daughter<br>Grandchild<br>Brother/sister<br>Father/mother<br>Father/mother of spouse<br>Child of relative<br>Child of non-relative<br>Other relative<br>Other non-relative<br>House help
2.6	What is [name]'s highest educational qualification?	Pre-primary<br>Primary<br>Post-primary, vocational<br>Secondary<br>College (middle level)<br>University undergraduate<br>University postgraduate<br>Madrassa/duksi<br>Other [list]
2.7	What is [name]'s working status?	Employed full-time (30+ hours per week)<br>Employed part time<br>Looking after home or family<br>Unemployed or on sick leave<br>Retired<br>Studying<br>Voluntary worker<br>Other [list]
2.8	What is [name]'s ethnicity?	Embu<br>Indian

		Kalenjin<br>Kamba<br>Kikuyu<br>Kisii<br>Luhya<br>Luo<br>Maasai<br>Meru<br>Somali<br>Half cast<br>Other [list]
	Now: Select up to five adult members (aged over 18 years) of the household to take part in the individual questionnaire and anthropometric measurements. If more than five are present, select the head of household and the person usually responsible for buying food for the household, then assign the rest in the order of who has the next birthday.<br><br>Now: Select up to five child members (aged 5 to 17 years) of the household to take part in the anthropometric measurements. If more than five are present, assign in the order of who has the next birthday.	[Assign individual ID to each adult, maximum of five]<br><br>[Assign individual ID to each child, maximum of five]
2.9	What type of main dwelling does the household live in?	Bungalow<br>Flat<br>Maisonette<br>Swahili<br>Shanty<br>Manyatta/traditional house<br>Other [list]
2.10	Does your household own this dwelling (house, flat, shack), do you rent it, or do you live here without pay?	Owns<br>Pays rent/lease<br>No rent, with consent of owner<br>No rent, squatting
2.11	Does this household have electricity?	Yes<br>No
2.12	What is the main source of water for your household?	Piped water (into dwelling or plot)<br>Public tap/stand pipe<br>Tubewell/borehole with pump

		Dug well<br>Water from spring<br>Rainwater collection<br>Vendors (e.g tanker or cart)<br>Surface water (e.g. river, stream, pond)<br>Bottled water<br>Other [list]
2.13	What is the main type of appliance used for cooking in your household?	Stone fire<br>Jiko<br>Kersone stove<br>Gas cooker<br>Electric cooker<br>Gas/electric cooker<br>Other [list]
2.14	What is the main source of energy for cooking in your household?	Firewood<br>Electricity<br>Liquefied petroleum gas (LPG)<br>Biogas<br>Kerosene<br>Charcoal<br>Straw/shrubs/grass<br>Animal dung<br>Agricultural crop residue<br>Other [list]
2.15	During the last 12 months, has any member of the household reared animals for consumption by the household (for example cows, goats, sheep, chickens or pigs)?	Yes<br>No
2.16	During the last 12 months, has any member of the household grown any food for consumption by the household (for example fruit or vegetables)?	Yes<br>No
2.17	Does this household have a refrigerator?	Yes<br>No
2.18	Does this household own a private car, or have one available for use?	Yes<br>No
2.19	[Confirm that all information about money and income will be kept strictly confidential].	Ksh<br><br>OR one of the following options:

	What is the current average monthly household income?<br><br>[Document only gross income (the entire amount of income before any deductions are made, such as tax) for employed participants.]	Less than 5,000 Ksh per month<br>5,000 to <10,000 Ksh per month<br>10,000 to <25,000 Ksh per month<br>25,000 to <40,000 Ksh per month<br>40,000 to <55,000 Ksh per month<br>55,000 or more Ksh per month<br><br>OR<br><br>Refused/don't know
**Section 3 Household food purchasing** [Interviewer to complete with person usually responsible for food purchasing]		
3.1	During the last month, what sources have been used to acquire food for the household?	Purchased<br>Own produce<br>Own stock<br>Gift<br>Relief<br>Payment in kind<br>Gathering/hunting/fishing<br>Barter<br>Other [list]
3.2	If any food has been purchased:<br>During the last month, what food retail outlets have been used to purchase food for the household?	Supermarket<br>Open air market<br>Kiosk<br>General shop<br>Specialised shop<br>Informal (roadside) vendor<br>Restaurant<br>Fast food<br>Café<br>Online<br>Other [list]
3.3	For each food retail outlet reported:<br>You mentioned that you bought from [food retail outlet] during the last month; what did you buy there?	Cereals/grain based foods<br>White roots, tubers and plantains<br>Pulses (beans, peas and lentils)<br>Nuts and seeds<br>Dairy (milk and milk products)<br>Meat and poultry

		Processed meat<br>Fish and seafood<br>Eggs<br>Vegetables<br>Fruit<br>Oils and fats<br>Savoury and fried snacks<br>Sweets<br>Herbs/spices/condiments<br>Alcoholic beverages<br>Non-alcoholic beverages (including tea, coffee etc.)<br>Ready-to-eat meals (precooked meals, prepared sandwiches, noodles etc.)<br>Other [list]
	If any food has been purchased from a supermarket:<br>During the last month, what supermarkets have been used to purchase food for the household?<br><br>[Try to get as much information about the supermarket as possible, including the name and address, so that it can be located later.]<br><br>Specific probe: During the last month, did you purchase food from the hypermarket at Lake Basin Mall in Kisumu? [If yes, add to list]	
3.4	Name of supermarket 1	[list]
3.5	Location/address of supermarket 1	[list]
3.6	Name of supermarket 2	[list]
3.7	Location/address of supermarket 2	[list]
3.8	Name of supermarket 3	[list]
3.9	Location/address of supermarket 3	[list]
3.10	During the last month, how much money was spent on food for the entire household?	Ksh
	Of the money spent on food for the entire household, how much was spent at a…..<br><br>[Make sure that that the values for questions 3.11 to 3.21 add to	

	the total value given in question 3.10.]	
3.11	Supermarket	Ksh
3.12	Open air market	Ksh
3.13	Kiosk	Ksh
3.14	General shop	Ksh
3.15	Specialised shop	Ksh
3.16	Informal (roadside) vendor	Ksh
3.17	Restaurant	Ksh
3.18	Fast food	Ksh
3.19	Café	Ksh
3.20	Online	Ksh
3.21	Other	Ksh
3.22	During the last month, what other costs were there when you did the food shopping?<br><br>[Include costs such as public transport, delivery charges, petrol, parking, and childcare). If there are no costs, tick None.]	Ksh<br>None
**Section 4 Travel to buy food** [Interviewer to complete with person usually responsible for food purchasing]		
4.1	During the last month, how many trips did you make to buy food for the household?<br><br>A trip is defined as a one-way course of travel with a single main purpose. If you travel from home to the store to buy food, then back home again, this counts at two trips.<br><br>[Make sure that that the values for questions 4.2 to 4.10 add to the total value given in question 4.1]	x trips
4.2	How many of these trips were made by foot?	x trips
4.3	How many of these trips were made by car?	x trips
4.4	How many of these trips were made by bicycle?	x trips
4.5	How many of these trips were made by public bus?	x trips
4.6	How many of these trips were made by matatu?	x trips
4.7	How many of these trips were made by boda boda?	x trips
4.8	How many of these trips were made by motorcycle?	x trips
4.9	How many of these trips were made by tuk tuk?	x trips

4.10	How many of these trips were made by other modes?	x trips
4.11	During the last month, how long did it usually take you to travel to the place where you do your main food shop?	x minutes
**Section 5 Food security** [Interviewer to complete with person usually responsible for food purchasing]		
5.1	In the last 12 months, did you or any household member eat fewer kinds of food due to lack of money or other resources?	Yes<br>No
5.2	In the last 12 months, did you or any household member miss a meal because of lack of money or other resources to obtain food?	Yes<br>No
5.3	In the last 12 months, did you worry that your household would not have enough food?	Yes<br>No
5.4	In the last 12 months were you or any household member not able to eat the kinds of food you preferred because of lack of money?	Yes<br>No
5.5	In the last 12 months, did you or any other household member eat less than you thought you should because of lack of money or other resources?	Yes<br>No
5.6	In the last 12 months, did your household run out of food because of lack of money or other resources?	Yes<br>No
5.7	In the last 12 months, were you or any other household member hungry but did not eat because of lack of money or other resources?	Yes<br>No
5.8	In the last 12 months, did you or any household member go without food for a whole day because of lack of money or other resources?	Yes<br>No
5.9	In the last 12 months, did the household receive any relief food?	Yes<br>No
**Section 6 Contact details** [Interviewer to complete with head of household]		
6.1	Could you please give us your cellphone number and/or at least two other family members/relatives/friends of your household such that we can contact you if we need more information?	Yes<br>No
6.2	Name 1	
6.3	Phone number 1	
6.4	Name 2	
6.5	Phone number 2	

6.6	Name 3	
6.7	Phone number 3	
6.8	There is another part to the study which involves participating in focus groups to talk about food, diet and health in the local community. Would you be willing to be contacted with more information about this? This part of the study is optional, and you do not have to agree to do it right now.	Yes<br>No
**Section 7: Anthropometric measurements for children**		
	Anthropometric measurements will be collected for a maximum of five children aged 5 to 17 years in the household. Remember to use the correct ID for each child from the start of the household survey.<br>Anthropometric measurements for adults will be collected as part of their individual questionnaire.<br><br>Remember to note the device ID for the stadiometer and scales you use. For each child, take one measurement of height and one measurement of weight. Take two measurements of waist circumference, and take a third measurement if there is a difference of ≥ 3cm between the first two measurements.	
7.1	Device ID for stadiometer	
7.2	Device ID for scales	
7.3	Child 1 individual ID	
7.4	Child 1 Height 1	xx cm
7.5	Child 1 Weight 1	xx kg
7.6	Child 1 Waist circumference 1	xx cm
7.7	Child 1 Waist circumference 2	xx cm
7.8	Child 1 Waist circumference 3	xx cm
7.9	Child 2 individual ID	
7.10	Child 2 Height 1	xx cm
7.11	Child 2 Weight 1	xx kg
7.12	Child 2 Waist circumference 1	xx cm
7.13	Child 2 Waist circumference 2	xx cm
7.14	Child 2 Waist circumference 3	xx cm
7.15	Child 3 individual ID	

7.16	Child 3 Height 1	xx cm
7.17	Child 3 Weight 1	xx kg
7.18	Child 3 Waist circumference 1	xx cm
7.19	Child 3 Waist circumference 2	xx cm
7.20	Child 3 Waist circumference 3	xx cm
7.21	Child 4 individual ID	
7.22	Child 4 Height 1	xx cm
7.23	Child 4 Weight 1	xx kg
7.24	Child 4 Waist circumference 1	xx cm
7.25	Child 4 Waist circumference 2	xx cm
7.26	Child 4 Waist circumference 3	xx cm
7.27	Child 5 individual ID	
7.28	Child 5 Height 1	xx cm
7.29	Child 5 Weight 1	xx kg
7.30	Child 5 Waist circumference 1	xx cm
7.31	Child 5 Waist circumference 2	xx cm
7.32	Child 5 Waist circumference 3	xx cm

Individual consent

Did you read and understand the information sheet and consent form? Can I explain anything? Do you have any questions? We would like to stress that we would not identify specific people in any write ups.

[Review participant information sheet and consent form and sign consent forms]

[Give a short introduction to the Hypermarket, Foodscape & Health study]

[Explain that in total the interview will take approximately one and a half hours; check how much time they have available]

[Explain that the first part of the household survey will be completed with the household head, and the second part with the person who is usually responsible for buying food for the household. There will also be a short individual survey for each member of the household aged over 18 years, limited to a maximum of five people. Finally, height, weight and waist circumference measurements will be taken from all those participating].

Supplementary Table S2 Individual survey.

#	Question	Answer
**Section 1: Interview details** [Interviewer to complete]		
1.1	Household ID	
1.2	Individual ID (MATCH WITH HOUSEHOLD SURVEY)	
1.3	Interviewer ID	
1.4	Completion	Interview completed<br>Interview partly completed
1.5	Visit 1 date	dd/mm/yyyy
1.6	Visit 1 time started	hh:mm
1.7	Visit 1 time ended	hh:mm
1.8	Visit 2 date	dd/mm/yyyy
1.9	Visit 2 time started	hh:mm
1.10	Visit 2 time ended	hh:mm
1.11	Visit 3 date	dd/mm/yyyy
1.12	Visit 3 time started	hh:mm
1.13	Visit 3 time ended	hh:mm
1.14	Main language of interview	English<br>Kiswahili<br>Luo
1.15	Supervisor signature	
1.16	Supervisor date	dd/mm/yyyy
1.17	Data entry signature	
1.18	Data entry date	dd/mm/yyyy
**Section 2: Demographics**		
2.1	How old are you?	xx years
2.2	What is your gender?	Male<br>Female
2.3	Has a doctor ever told you that you have raised or high blood pressure or hypertension?<br><br>[Exclude hypertension during pregnancy]	Yes<br>No<br>Don't know
2.4	If yes to high blood pressure:<br>Are you currently prescribed medication for this?	Yes<br>No

2.5	Has a doctor ever told you that you have diabetes?<br><br>[Exclude gestational diabetes]	Yes<br>No<br>Don't know
2.6	If yes to diabetes:<br>Are you currently prescribed medication for this, including insulin?	Yes<br>No
2.7	If yes to diabetes medication:<br>What medication have you been prescribed?	Insulin<br>Oral medication<br>Both
2.8	Has a doctor ever told you that you have raised blood cholesterol?	Yes<br>No<br>Don't know
2.9	Has a doctor ever told you that you have heart disease?	Yes<br>No<br>Don't know
2.10	Has a doctor ever told you that you have had a stroke?	Yes<br>No<br>Don't know
2.11	Has a doctor ever told you that you have cancer?	Yes<br>No<br>Don't know
**Section 3: 24 hour diet recall**		
3.1	To begin, I'd like to get an idea of the type of food and drink you had yesterday.<br>With regard to what you ate, was yesterday a typical/usual day for you?	Yes<br>No
3.2	[If yes] Please describe how it was unusual?	Any celebration<br>Fasting<br>Unwell<br>Funeral<br>Other [list]
	Please describe ALL the foods (meals and snacks) that you ate or drank yesterday during the day and night, whether at home or outside the home. Start with the first food or drink eaten in the morning and indicate the approximate time you ate or drank. Remember to include any sauces, dressings or accompaniments that you consumed with your meals or snacks.	

	I'd also like you to tell me where this food/drink was purchased, where it was prepared and where it was consumed. For example:<br><br>Purchased out of home, but prepared and consumed at home (e.g. cooking and eating a meal at home, using ingredients purchased from the supermarket).<br><br>Purchased and prepared out of home, but consumed at home (e.g. buying hot food and bringing it home to eat).<br><br>Purchased, prepared and consumed out of home (e.g. eating at a restaurant)<br><br>I'd like you to tell me about foods you ate even in small amounts less than 15 grams (use showcard).	
3.3	Time 1	hh:mm
3.4	Food/drink consumed	[Free text]
3.5	Quantity consumed less than 15g	Tick
3.6	Where was this food/drink purchased?	[List]<br>Food not purchased
3.7	Where was this food/drink prepared?	In home<br>Out of home
3.8	Where was this food/drink consumed?	In home<br>Out of home
3.9	Time 2	hh:mm
3.10	Food/drink consumed	[Free text]
3.11	Quantity consumed less than 15g	Tick
3.12	Where was this food/drink purchased?	[List]<br>Food not purchased
3.13	Where was this food/drink prepared?	In home<br>Out of home
3.14	Where was this food/drink consumed?	In home<br>Out of home
3.15	Time 3	hh:mm
3.16	Food/drink consumed	[Free text]

3.17	Quantity consumed less than 15g	Tick
3.18	Where was this food/drink purchased?	[List]<br>Food not purchased
3.19	Where was this food/drink prepared?	In home<br>Out of home
3.20	Where was this food/drink consumed?	In home<br>Out of home
3.21	Time 4	hh:mm
3.22	Food/drink consumed	[Free text]
3.23	Quantity consumed less than 15g	Tick
3.24	Where was this food/drink purchased?	[List]<br>Food not purchased
3.25	Where was this food/drink prepared?	In home<br>Out of home
3.26	Where was this food/drink consumed?	In home<br>Out of home
3.27	Time 5	hh:mm
3.28	Food/drink consumed	[Free text]
3.29	Quantity consumed less than 15g	Tick
3.30	Where was this food/drink purchased?	[List]<br>Food not purchased
3.31	Where was this food/drink prepared?	In home<br>Out of home
3.32	Where was this food/drink consumed?	In home<br>Out of home
3.33	Time 6	hh:mm
3.34	Food/drink consumed	[Free text]
3.35	Quantity consumed less than 15g	Tick
3.36	Where was this food/drink purchased?	[List]<br>Food not purchased
3.37	Where was this food/drink prepared?	In home<br>Out of home
3.38	Where was this food/drink consumed?	In home<br>Out of home
3.39	Time 7	hh:mm

3.40	Food/drink consumed	[Free text]
3.41	Quantity consumed less than 15g	Tick
3.42	Where was this food/drink purchased?	[List]<br>Food not purchased
3.43	Where was this food/drink prepared?	In home<br>Out of home
3.44	Where was this food/drink consumed?	In home<br>Out of home
3.45	Time 8	hh:mm
3.46	Food/drink consumed	[Free text]
3.47	Quantity consumed less than 15g	Tick
3.48	Where was this food/drink purchased?	[List]<br>Food not purchased
3.49	Where was this food/drink prepared?	In home<br>Out of home
3.50	Where was this food/drink consumed?	In home<br>Out of home
3.51	Time 9	hh:mm
3.52	Food/drink consumed	[Free text]
3.53	Quantity consumed less than 15g	Tick
3.54	Where was this food/drink purchased?	[List]<br>Food not purchased
3.55	Where was this food/drink prepared?	In home<br>Out of home
3.56	Where was this food/drink consumed?	In home<br>Out of home
3.57	Time 10	hh:mm
3.58	Food/drink consumed	[Free text]
3.59	Quantity consumed less than 15g	Tick
3.60	Where was this food/drink purchased?	[List]<br>Food not purchased
3.61	Where was this food/drink prepared?	In home<br>Out of home
3.62	Where was this food/drink consumed?	In home<br>Out of home

	When the participant has finished their recall, ask the following question to confirm the ingredients in any in any of the dishes reported and record in the boxes above.<br><br>Can you list the main ingredients in the [dish/food] that you ate yesterday?	
	Finally, ask these probing questions for any gaps in the food groups or times reported. Record any additional food/drink above.<br><br>Can you think of anything else that you ate or drank between meals or before bed?<br>Did you have any accompaniments or drinks with your meal or food?<br>Did you use or add any oils, fats, sweeteners or sugar when you were cooking, preparing or eating your meals (e.g. adding oil to salad or sugar to tea/coffee) ?<br>[If there are long gaps between foods] Can you remember if you ate anything at [meal/time] yesterday?<br>[If there are long gaps between foods] Can you remember if you ate anything between [meal/time] and [meal/time] yesterday?	
	Can you think of any food or ingredient that you ate yesterday which was less than a tablespoon (15g)? [Tick against 15g question, or add to list and tick if not recalled previously].	
	I am going to read what I have written back to you. Please let me know if there are any mistakes or additional food/drinks you can remember. If you ate less than a tablespoon of any of these foods, please could you indicate that too.<br><br>[The aim of this question is to confirm the recall whilst categorising and recording the food groups consumed. Indicate yes/no for each food group (i.e. yes if something was consumed from that group) in the matrix below. Please use the 'Food List' provided].]	
3.63	Foods made from grains	Yes > 15g<br>Yes 15g or less<br>No
3.64	White roots, tubers and plantains	Yes > 15g<br>Yes 15g or less<br>No

3.65	Pulses (beans, peas and lentils)	Yes > 15g<br>Yes 15g or less<br>No
3.66	Nuts and seeds	Yes > 15g<br>Yes 15g or less<br>No
3.67	Milk and milk products	Yes > 15g<br>Yes 15g or less<br>No
3.68	Organ meat	Yes > 15g<br>Yes 15g or less<br>No
3.69.1	Processed red meat	Yes > 15g<br>Yes 15g or less<br>No
3.69.2	Processed poultry	Yes > 15g<br>Yes 15g or less<br>No
3.69.3	Unprocessed red meat	Yes > 15g<br>Yes 15g or less<br>No
3.69.4	Unprocessed poultry	Yes > 15g<br>Yes 15g or less<br>No
3.70	Fish and seafood	Yes > 15g<br>Yes 15g or less<br>No
3.71	Eggs	Yes > 15g<br>Yes 15g or less<br>No
3.72	Dark green leafy vegetables	Yes > 15g<br>Yes 15g or less<br>No
3.73	Vitamin A rich vegetables and tubers	Yes > 15g<br>Yes 15g or less<br>No

3.74	Vitamin A rich fruits	Yes > 15g<br>Yes 15g or less<br>No
3.75	Other vegetable	Yes > 15g<br>Yes 15g or less<br>No
3.76	Other fruits	Yes > 15g<br>Yes 15g or less<br>No
3.77	Insects and other small protein foods	Yes > 15g<br>Yes 15g or less<br>No
3.78	Red palm oil	Yes > 15g<br>Yes 15g or less<br>No
3.79	Other oils and fats	Yes > 15g<br>Yes 15g or less<br>No
3.80	Savoury and fried snacks	Yes > 15g<br>Yes 15g or less<br>No
3.81	Sweets	Yes > 15g<br>Yes 15g or less<br>No
3.82	Sugar-sweetened beverages	Yes > 15g<br>Yes 15g or less<br>No
3.83	Condiments and seasonings	Yes > 15g<br>Yes 15g or less<br>No
3.84	Other beverages and foods	Yes > 15g<br>Yes 15g or less<br>No
	Refer to any food groups that weren't consumed. Record additional foods on the diet recall sheet and the food matrix.<br>Can you tell me if you ate any [food group] foods yesterday, such as [examples]?	

Section 4: Physical activity (GPAQ)		
	Next I am going to ask you about the time you spend doing different types of physical activity in a typical week. Please answer these questions even if you do not consider yourself to be a physically active person. Think first about the time you spend doing work. Think of work as the things that you have to do such as paid or unpaid work, study/training, household chores, harvesting food/crops, fishing or hunting for food, seeking employment. [Insert other examples if needed, use showcard]. In answering the following questions 'vigorous-intensity activities' are activities that require hard physical effort and cause large increases in breathing or heart rate, 'moderate-intensity activities' are activities that require moderate physical effort and cause small increases in breathing or heart rate.	
4.1	Does your work involve vigorous-intensity activity that causes large increases in breathing or heart rate like [carrying or lifting heavy loads, digging or construction work] for at least 10 minutes continuously?	Yes<br>No
4.2	If yes: In a typical week, on how many days do you do vigorous-intensity activities as part of your work?	xx days a week
4.3	If yes: How much time do you spend doing vigorous-intensity activities at work on a typical day?	hh:mm
4.4	Does your work involve moderate-intensity activity, that causes small increases in breathing or heart rate such as brisk walking [or carrying light loads] for at least 10 minutes continuously?	Yes<br>No
4.5	If yes: In a typical week, on how many days do you do moderate-intensity activities as part of your work?	xx days a week
4.6	If yes: How much time do you spend doing moderate-intensity activities at work on a typical day?	hh:mm
	The next questions exclude the physical activities at work that you have already mentioned. Now I would like to ask you about the usual way you travel to and from places. For example to work, for shopping, to market, to place of worship. [insert other examples if needed]	
4.7	Do you walk or use a bicycle (pedal cycle) for at least 10 minutes continuously to get to and from places?	Yes<br>No
4.8	If yes: In a typical week, on how many days do you walk or bicycle for at least 10 minutes continuously to get to and from places?	xx days a week
4.9	If yes: How much time do you spend walking or bicycling for travel on a typical day?	hh:mm

	The next questions exclude the work and transport activities that you have already mentioned. Now I would like to ask you about sports, fitness and recreational activities (leisure),[insert relevant terms, use showcard]	
4.10	Do you do any vigorous-intensity sports, fitness or recreational (leisure) activities that cause large increases in breathing or heart rate like [running or football,] for at least 10 minutes continuously?	Yes<br>No
4.11	If yes: In a typical week, on how many days do you do vigorous-intensity sports, fitness or recreational (leisure) activities?	xx days a week
4.12	If yes: How much time do you spend doing vigorous-intensity sports, fitness or recreational activities on a typical day?	hh:mm
4.13	Do you do any moderate-intensity sports, fitness or recreational (leisure) activities that causes a small increase in breathing or heart rate such as brisk walking,[cycling, swimming, volleyball] for at least 10 minutes continuously?	Yes<br>No
4.14	In a typical week, on how many days do you do moderate-intensity sports, fitness or recreational (leisure) activities?	xx days a week
4.15	How much time do you spend doing moderate-intensity sports, fitness or recreational (leisure) activities on a typical day?	hh:mm
	The following question is about sitting or reclining at work, at home, getting to and from places, or with friends including time spent [sitting at a desk, sitting with friends, travelling in car, bus, train, reading, playing cards or watching television], but do not include time spent sleeping.	
4.16	How much time do you usually spend sitting or reclining on a typical day?	hh:mm
**Section 5: Wellbeing (WHOQOL-BREF)**		
	This assessment asks how you feel about your quality of life, health, or other areas of your life. Please answer all the questions. If you are unsure about which response to give to a question, please choose the one that appears most appropriate. This can often be your first response.<br>Please keep in mind your standards, hopes, pleasures and concerns. We ask that you think about your life in the last two weeks.	
5.1	How would you rate your quality of life?	Very poor<br>Poor<br>Neither poor nor good

		Good<br>Very good
5.2	How satisfied are you with your health?	Very dissatisfied<br>Dissatisfied<br>Neither satisfied nor dissatisfied<br>Satisfied<br>Very satisfied
**Section 6: Social connectedness**		
	Next I'm going to ask you about your relationship with other people. Please tell me how much each statement describes your situation by using these responses.	
6.1	I know that people in my life accept and value me	1 very inaccurate<br>2<br>3<br>4<br>5<br>6<br>7 very accurate
6.2	I know that people around me share my attitudes and beliefs	1 very inaccurate<br>2<br>3<br>4<br>5<br>6<br>7 very accurate
6.3	I feel like an outsider	1 very inaccurate<br>2<br>3<br>4<br>5<br>6<br>7 very accurate
**Section 7: Anthropometric measurements**		
	Remember to note the device ID for the stadiometer and scales you use. For each participant, take one measurement of height and one measurement of weight. Take two measurements of waist	

	circumference, and take a third measurement if there is a difference of ≥ 3cm between the first two measurements.	
7.1	Device ID for stadiometer	
7.2	Device ID for scales	
7.3	Height 1	xx cm
7.4	Weight 1	xx kg
7.5	Waist circumference 1	xx cm
7.6	Waist circumference 2	xx cm
7.7	Waist circumference 3	xx cm

Supplementary Table S3 Diet recall sheet used alongside individual survey.

Diet recall sheet

**Household ID**	
**Individual ID**	

For each food and drink, I'd also like you to tell me where this food/drink was purchased, where it was prepared and where it was consumed.

Examples: Purchased out of home, but prepared and consumed at home (e.g. cooking and eating a meal at home, using ingredients purchased from the supermarket). Purchased and prepared out of home, but consumed at home (e.g. buying hot food and bringing it home to eat). Purchased, prepared and consumed out of home (e.g. eating at a restaurant).

For pre-prepared or packaged foods, tick 'not applicable' for the question on where food//drink was prepared.

Time	Food/drink consumed (one food/drink per box)	Quantity consumed less than 15g? (use showcard)	Where was this food/drink purchased?	Where was this food/drink prepared?	Where was this food/drink consumed?
		Yes ☐ No ☐	Not purchased ☐<br>---------------------------	In home ☐<br>Out of home ☐<br>Not applicable ☐	In home ☐<br>Out of home ☐
		Yes ☐ No ☐	Not purchased ☐<br>---------------------------	In home ☐<br>Out of home ☐<br>Not applicable ☐	In home ☐<br>Out of home ☐
		Yes ☐ No ☐	Not purchased ☐<br>---------------------------	In home ☐<br>Out of home ☐<br>Not applicable ☐	In home ☐<br>Out of home ☐
		Yes ☐ No ☐	Not purchased ☐<br>---------------------------	In home ☐<br>Out of home ☐<br>☐	In home ☐<br>Out of home ☐

				Not applicable	
		Yes No	Not purchased<br>----------------------------	In home<br>Out of home<br>Not applicable	In home<br>Out of home
		Yes No	Not purchased<br>----------------------------	In home<br>Out of home<br>Not applicable	In home<br>Out of home
		Yes No	Not purchased<br>----------------------------	In home<br>Out of home<br>Not applicable	In home<br>Out of home
		Yes No	Not purchased<br>----------------------------	In home<br>Out of home<br>Not applicable	In home<br>Out of home
		Yes No	Not purchased<br>----------------------------	In home<br>Out of home<br>Not applicable	In home<br>Out of home
		Yes No	Not purchased<br>----------------------------	In home<br>Out of home<br>Not applicable	In home<br>Out of home
		Yes No	Not purchased<br>----------------------------	In home<br>Out of home<br>Not applicable	In home<br>Out of home

		Yes ☐ No ☐	Not purchased ☐<br>----------------------------	In home ☐<br>Out of home ☐<br>Not applicable ☐	In home ☐<br>Out of home ☐
		Yes ☐ No ☐	Not purchased ☐<br>----------------------------	In home ☐<br>Out of home ☐<br>Not applicable ☐	In home ☐<br>Out of home ☐
		Yes ☐ No ☐	Not purchased ☐<br>----------------------------	In home ☐<br>Out of home ☐<br>Not applicable ☐	In home ☐<br>Out of home ☐
		Yes ☐ No ☐	Not purchased ☐<br>----------------------------	In home ☐<br>Out of home ☐<br>Not applicable ☐	In home ☐<br>Out of home ☐
		Yes ☐ No ☐	Not purchased ☐<br>----------------------------	In home ☐<br>Out of home ☐<br>Not applicable ☐	In home ☐<br>Out of home ☐
		Yes ☐ No ☐	Not purchased ☐<br>----------------------------	In home ☐<br>Out of home ☐<br>Not applicable ☐	In home ☐<br>Out of home ☐
		Yes ☐ No ☐	Not purchased ☐<br>----------------------------	In home ☐<br>Out of home ☐<br>Not applicable ☐	In home ☐<br>Out of home ☐
		Yes ☐ No ☐	Not purchased ☐	In home ☐	In home ☐

			----------------------------	Out of home<br>Not applicable	Out of home
		Yes No	Not purchased<br>----------------------------	In home<br>Out of home<br>Not applicable	In home<br>Out of home
		Yes No	Not purchased<br>----------------------------	In home<br>Out of home<br>Not applicable	In home<br>Out of home
		Yes No	Not purchased<br>----------------------------	In home<br>Out of home<br>Not applicable	In home<br>Out of home
		Yes No	Not purchased<br>----------------------------	In home<br>Out of home<br>Not applicable	In home<br>Out of home
		Yes No	Not purchased<br>----------------------------	In home<br>Out of home<br>Not applicable	In home<br>Out of home
		Yes No	Not purchased<br>----------------------------	In home<br>Out of home<br>Not applicable	In home<br>Out of home
		Yes No	Not purchased<br>----------------------------	In home<br>Out of home	In home<br>Out of home

				Not applicable	
		Yes No	Not purchased<br>----------------------------	In home<br>Out of home<br>Not applicable	In home<br>Out of home
		Yes No	Not purchased<br>----------------------------	In home<br>Out of home<br>Not applicable	In home<br>Out of home
		Yes No	Not purchased<br>----------------------------	In home<br>Out of home<br>Not applicable	In home<br>Out of home
		Yes No	Not purchased<br>----------------------------	In home<br>Out of home<br>Not applicable	In home<br>Out of home
		Yes No	Not purchased<br>----------------------------	In home<br>Out of home<br>Not applicable	In home<br>Out of home
		Yes No	Not purchased<br>----------------------------	In home<br>Out of home<br>Not applicable	In home<br>Out of home
		Yes No	Not purchased<br>----------------------------	In home<br>Out of home<br>Not applicable	In home<br>Out of home

		Yes ☐ No ☐	Not purchased ☐<br>----------------------------	In home ☐<br>Out of home ☐<br>Not applicable ☐	In home ☐<br>Out of home ☐
		Yes ☐ No ☐	Not purchased ☐<br>----------------------------	In home ☐<br>Out of home ☐<br>Not applicable ☐	In home ☐<br>Out of home ☐
		Yes ☐ No ☐	Not purchased ☐<br>----------------------------	In home ☐<br>Out of home ☐<br>Not applicable ☐	In home ☐<br>Out of home ☐
		Yes ☐ No ☐	Not purchased ☐<br>----------------------------	In home ☐<br>Out of home ☐<br>Not applicable ☐	In home ☐<br>Out of home ☐
		Yes ☐ No ☐	Not purchased ☐<br>----------------------------	In home ☐<br>Out of home ☐<br>Not applicable ☐	In home ☐<br>Out of home ☐
		Yes ☐ No ☐	Not purchased ☐<br>----------------------------	In home ☐<br>Out of home ☐<br>Not applicable ☐	In home ☐<br>Out of home ☐
		Yes ☐ No ☐	Not purchased ☐<br>----------------------------	In home ☐<br>Out of home ☐<br>Not applicable ☐	In home ☐<br>Out of home ☐
		Yes ☐ No ☐	Not purchased ☐	In home ☐	In home ☐

			----------------------------	Out of home<br>Not applicable ☐	Out of home
		Yes ☐ No ☐	Not purchased ☐<br>----------------------------	In home ☐<br>Out of home ☐<br>Not applicable ☐	In home ☐<br>Out of home ☐
		Yes ☐ No ☐	Not purchased ☐<br>----------------------------	In home ☐<br>Out of home ☐<br>Not applicable ☐	In home ☐<br>Out of home ☐
		Yes ☐ No ☐	Not purchased ☐<br>----------------------------	In home ☐<br>Out of home ☐<br>Not applicable ☐	In home ☐<br>Out of home ☐
		Yes ☐ No ☐	Not purchased ☐<br>----------------------------	In home ☐<br>Out of home ☐<br>Not applicable ☐	In home ☐<br>Out of home ☐
		Yes ☐ No ☐	Not purchased ☐<br>----------------------------	In home ☐<br>Out of home ☐<br>Not applicable ☐	In home ☐<br>Out of home ☐
		Yes ☐ No ☐	Not purchased ☐<br>----------------------------	In home ☐<br>Out of home ☐<br>Not applicable ☐	In home ☐<br>Out of home ☐
		Yes ☐ No ☐	Not purchased ☐<br>----------------------------	In home ☐<br>Out of home ☐ ☐	In home ☐<br>Out of home ☐

				Not applicable	
		Yes No	Not purchased<br>---------------------------	In home<br>Out of home<br>Not applicable	In home<br>Out of home
		Yes No	Not purchased<br>---------------------------	In home<br>Out of home<br>Not applicable	In home<br>Out of home
		Yes No	Not purchased<br>---------------------------	In home<br>Out of home<br>Not applicable	In home<br>Out of home
		Yes No	Not purchased<br>---------------------------	In home<br>Out of home<br>Not applicable	In home<br>Out of home

Supplementary Table S4 Questions from the household survey used to ascertain household food insecurity.

**Question wording used in household survey**	**FIES question wording**
In the last 12 months, did you worry that your household would not have enough food?	During the last 12 MONTHS, was there a time when you were worried you would not have enough food to eat because of a lack of money or other resources?
In the last 12 months were you or any household member not able to eat the kinds of food you preferred because of lack of money?	Still thinking about the last 12 MONTHS, was there a time when you were unable to eat healthy and nutritious food because of a lack of money or other resources?
In the last 12 months, did you or any household member eat fewer kinds of food due to lack of money or other resources?	Was there a time when you ate only a few kinds of foods because of a lack of money or other resources?
In the last 12 months, did you or any household member miss a meal because of lack of money or other resources to obtain food?	Was there a time when you had to skip a meal because there was not enough money or other resources to get food?
In the last 12 months, did you or any other household member eat less than you thought you should because of lack of money or other resources?	Still thinking about the last 12 MONTHS, was there a time when you ate less than you thought you should because of a lack of money or other resources?
In the last 12 months, did your household run out of food because of lack of money or other resources?	Was there a time when your household ran out of food because of a lack of money or other resources?
In the last 12 months, were you or any other household member hungry but did not eat because of lack of money or other resources?	Was there a time when you were hungry but did not eat because there was not enough money or other resources for food?
In the last 12 months, did you or any household member go without food for a whole day because of lack of money or other resources?	During the last 12 MONTHS, was there a time when you went without eating for a whole day because of a lack of money or other resources?
In the last 12 months, did the household receive any relief food?	

Agriculture Organisation's Food Insecurity Experience Scale (FIES)

Supplementary Table S5 Food list used by fieldworkers to classify consumed items from the diet recall into appropriate food groups

Code	Food group	Example food/drink
A	Foods made from grains	Corn/maize, rice (e.g pishori), wheat, oats, sorghum, millet (e.g. wimbi), barley or any other grains or foods made from these grains (e.g. bread and flatbreads, wheat buns/scones, noodles (e.g. indomie), pasta (e.g. spaghetti, macaroni), porridge, ugali, nshima, roti, farine, saltbread, pizza
B	White roots, tubers and plantains	White (Irish) potatoes, white yam, white cassava, tapioca, plantain/yudi, arrowroot (nduma), taro (dalo/dasheen/giant taro), eddo, yautia (tannia/malanga), yam (yampi), yucca, yambean, dalonitana, Tivoli, Kawai (sweet yam), sweet potato (white flesh), breadfruit.
C	Pulses (beans, peas and lentils)	Any bean, pea or lentil such as: common bean (black, kidney, pinto), dolichos/njahi black beans, split peas, katanaku, lima, mung (e.g. green gram), soy, pigeon pea, bodi bean, wingbean, broad bean (fava, field bean), chickpea or chicken pea (garbanzo), pigeon pea, cowpea, lentil and soybean. Includes products such as tofu, tempeh and other processed soy/soybean, lentil or legume products. Also any bean consumed as hummus.
D	Nuts and seeds	Nuts, seeds or foods made from these (eg.peanut butter). Betel nut, breadfruit seeds, candlenut kernels, pandanus nuts, jackfruit seeds, pili nut, veitchia nut, cottonseed meal, lupins, breadnut, peanut/groundnut, cashew, macadamia, brazil nut, almond, chestnut, hazelnut, pecan, pistachio, walnut, sesame seed (e.g. simsim), sunflower seed, pumpkin/squash/gourd seed, pine nut and coconut (not milk/butter classed as ‘other oils and fats’).
E	Milk and milk products	Milk, cheese, yogurt/curd or other milk products. Includes, fresh, powdered, evaporated or UHT milk, cow's milk, goat's milk, camel’s milk, almond milk, soy milk. Hard cheese (e.g. cheddar, mozzarella, Swiss, parmesan). Soft cheese (e.g. ricotta, cottage, paneer). Kefir. Milk sour (mala).
F	Organ meat	Liver (including liver served as pate), kidney, heart, gizzard, or other organ meats or blood-based foods such as black sausage/black pudding.
G	Meat and poultry	
G1	Processed red meat	Ham, sausage, hot dog, smokies, burger, camp pie, bologna sausage, Devon (sausage)/fritz, beef or pork brawn, bacon, salami, canned beef, meat paste for hamburger, aliya (salted sun dried meat)
G2	Processed poultry	Chicken nuggets, turkey sausage
G3	Unprocessed red meat	Beef, minced meat, pork (pig), lamb, sheep, goat, camel, rabbit, mutton flaps, dog, bat, deer, crocodile, horse, mongoose, manicou (possum), iguana, turtle, tripe (matumbo), hippo, gazelle
G4	Unprocessed poultry	Chicken (e.g. broiler, kienyeji) , duck, goose, guinea fowl, turkey, pigeon, quail
H	Fish and seafood	Fresh, frozen, dried or canned fish or seafood, large or small, all species. Such as: omena, tilapia, nile perch, anchovies, tuna, salmon, sardines, clams, mussels, oysters and scallops, shrimp, lobster, crayfish and crabs, prawns, edible sea urchins and sea cucumbers, octopus, squid and cuttlefish, shark, whale, fried flying fish, frizzled salt fish, blue marlin, seasnail, wahoo, banga-marie, nama, lumi, sea cucumber, jacks, robin fish, king fish, blue marlin, swordfish, black fish, red snapper, cavalli, bonita, baracuda, balahoo, tri tri, conch, sea eggs, eel (sea snake), whelks.
I	Eggs	Any egg such as goose eggs, duck eggs, turkey eggs, chicken (kienyenji) eggs, guinea fowl or any other egg (including those in omelette).

J	Dark green leafy vegetables	Amaranth leaves, cassava leaves, kale, collard greens (sukuma wiki), spider plant/spider web (sagaa or saget), slender leaf (mitoo), apoth, night shade, spinach, Silverbeet, taro/daro stalk, rourou (taro/daro leaves), kangkong, bele (aibika, aelan cabbage, slippery cabbage or pele), tubua, beans leaves, Chinese cabbage, romaine lettuce, bibb lettuce (NOT iceberg lettuce), jointfir leaves, nightshade leaves, drumstrick leaves, chocko leaves, saijan leaves, bele sikau, dasheen leaves and stalks, pumpkin leaves, watercress, patchoi.
K	Vitamin A rich vegetables and tubers	Orange/yellow-fleshed sweet potato/kumala, carrot, pumpkin, yellow or orange fleshed squash (e.g. butternut), red sweet pepper (red capsicum/pilipili hoho), red chilli pepper, yellow-fleshed taro
L	Vitamin A rich fruits	Fresh or dried versions of the following: Ripe mango, ripe papaya (pawpaw), red palm fruit, passion fruit (granadilla), apricot, yellow or orange fleshed bananas, cantaloupe melon, musk melon, hog plum, locust bean/pulp, loquat, peach, persimmon, pitanga, tree tomato (tamarillo).
M	Other vegetable	Tomato, cucumber, onion, leek, celery, yellow or green capsicum (pilipili hoho), baigan/eggplant/aubergine (biringanya), courgette/zucchini, marrow, beetroot, light green lettuce (iceberg), artichoke, gourd, okra, broccoli, endive, prickly pear, jackfruit, ota (fern), lauki (bottle gourd/calabash), duruka, carailla, sweet/bitter gourd/karela, chichira, sweet corn, baby corn, mushroom, fresh peas eaten as fresh pod such as garden peas, snow peas, snap peas, french beans, runner beans or green beans.
N	Other fruits	All fresh or dried fruits NOT mentioned in Vitamin A rich fruits, but not sugar sweetened processed fruit products (which are considered as 'sweets'): Green mango, green papaya (pawpaw), white fleshed bananas (not plantain), apple, avocado, karat, pandanus, garlic-pear fruit (yellow fleshed), golden apple. mamee apple (mamsiport), chinese apple, Sapodilla, Soursop, Sapote, Tamarind, Tarawau, Chayote (chocho/chocko/christophene/pear squash) Jambolan (java plum/jamun), pomelo (shaddock), Dawa (fijian longan), Kavika (rose apple), Moli (orange/mandarin), guava, pomegranate, tangerine, orange, grapefruit, strawberries, grapes, pineapples, custard apple, carambola, wax apple, plum rose, bequia plum, fat pork, sea side grapes, goose berries or damsel, cherries, sweet sopbell, melons (except cantaloupe), jackfruit.
O	Insects and other small protein foods	Fish roe, insects (locust, termite), insect eggs, insect larvae/grubs, snails, spiders, any small invertebrates. (Note that this category does NOT include frogs, snakes, or other reptiles/amphibians which are included in 'meat and poultry').
P	Red palm oil	Red palm oil
Q	Other oils and fats	Butter, cream and sour cream, ghee, lard, suet, tallow, margarine, mayonnaise, palm oil, cooking fat, cooking oil, or any oil extracted form fruit/vegetable/nut/seed or grain (canola, coconut, cottonseed, groundnut, maize, olives, rapeseed, safflower, sesame, soybean, sunflowers, walnut).
R	Savoury and fried snacks	Cassava chips, fried cassava balls, other cassava-based fried snacks, corn/maize chips/fried tortilla strips, potato/sweet potato chips, crisps, puffs (cheese/corn/maize or other 'puffs'), doughnuts/fry bread, samosas, mandazi, other deep-fried, mainly carbohydrate snack/processed street food. (Fried potatoes or fried plantains should NOT be classified here. Classify under 'White roots, tubers and plantains').
S	Sweets	Any foods with a with a high content of different sweetening agents (e.g. sugar, sugar cane, corn syrup, other syrup, honey, molasses or jiggery (nguru)) such as: Sweet biscuits/cookies, sugar icing, cakes, pastries, sweet pies, candies, chocolate, sugar sweetened chewing gum, sweetened coconut snacks, custard, canned fruit in sugar syrup, honey, ice cream, sweetened condensed milk, jams, marmalade

T	Sugar-sweetened beverages	All sugar-sweetened beverages, with any/all other ingredients. Examples include: tea, coffee WITH sugar (if without sugar then classified under 'Group V Other beverages'), chocolate/cocoa drinks, energy/sports drinks, fruit drinks/sweetened fruit juices/squash (unless certain that the juice was made from 100% fruit with no added sweetener – if 100% fruit juice then classify under Group L or N depending on the type of fruit), malt drinks (fortified and unfortified), soft drinks/sodas/carbonated or 'fizzy drinks' including colas, fruit and other flavours (if diet soft drinks, then classified under 'Group V Other beverages'), yogurt drinks, condensed milk (if diluted and consumed as a drink), sweetened milkshakes, any other drink sweetened with sugar, corn syrup, honey or other sweetener.
U	Condiments and seasonings	Any herb, spice or other seasoning/condiment that is added for flavor in small amounts such as, kava, lemon juice/moli, lime juice, vinegar, shallot, onion, garlic, salt, magadi (sodium bicarbonate), soy sauce, tamari, hot/chilli sauce, fish sauce/powder, ginger root (tangawizi), horseradish, yeast, all types of fresh or dried herbs (e.g. coriander/ dhania, sage, thyme, lime, basil, chive, shadon beni), spices dried or fresh (e.g. chilli/rokete, black pepper, paprika, pilau masala), ketchup/tomato sauce, mustard, pepper sauce, seeds or seed pastes (when used to flavour or garnish), sugar (when added to flavour a mixed dish), tomato paste, dried soup seasoning packets, any other seasoning or flavouring added during cooking (e.g. Royco, Knorr), any garnish added at the end of cooking or when serving.
V	Other beverages and foods	Any other food or beverage not in groups A-U. For example, alcohol, diet soft drinks, chutney/pickle/pickled cucumbers, clear broth/soup broth, coffee or tea (with or without milk) if unsweetened, herbal beverage/infusions, olives.

Supplementary Table S6 Characteristics of households with and without missing data on household head

	Households with missing household head data (n=8) n (%)	Households without missing household head data (n=368) n (%)	P-value for difference between households reporting and not reporting all household head data**
**Study area (n=376)**			
**Kisumu**	0 (0)	180 (49)	0.008*
**Household size (n=376)**			
**Median / IQR**	4 / 2.5	4 / 3	0.12
**Length of residence in the local area (years, n=376)**			
**Median / IQR**	30 / 34	12 / 22	0.086
**Type of dwelling (n=376)**			0.096
**Bungalow**	1 (13)	48 (13)	
**Flat**	4 (50)	36 (10)	
**Maisonette**	0	18 (5)	
**Swahili**	0	21 (6)	
**Shanty**	3 (37)	152 (41)	
**Manyatta/traditional house**	0	55 (15)	
**Other**	0	38 (10)	
**Electricity? (n=376)**			
**Yes**	3 (38)	238 (65)	0.14
**Main water source (n=376)**			0.015*
**Piped water**	2 (25)	88 (24)	
**Public tap/stand pipe**	0	157 (43)	
**Well**	1 (13)	26 (7)	
**Vendors**	3 (37)	30 (8)	
**Surface water**	2 (25)	59 (16)	
**Other**	0	8 (2)	
**Own fridge? (n=376)**			
**Yes**	2 (25)	56 (15)	0.36
**Own car? (n=376)**			
**Yes**	2 (25)	34 (9)	0.17

Note: results presented as n (%) unless otherwise indicated
*significant at the 0.05 level
**Fisher's exact test or Wilcoxon rank sum test was used to determine statistical differences between households reporting all household head data

Supplementary Table S7 Malnutrition characteristics for individuals included in analyses

	**Overall n (%)**	**Kisumu n (%)**	**Homabay n (%)**
Underweight BMI (n=492, 356 clusters)	51 (10)	31 (12)	20 (8)
Overweight/obese BMI (n=492, 356 clusters)	210 (43)	111 (43)	99 (42)
Insufficient micronutrient adequacy* (n=493, 357 clusters)	274 (56)	131 (51)	143 (60)

*Based on the Minimum Dietary Diversity for Women indicator

Supplementary Table S8 Number and proportion of 357 households (from which individual participants were drawn) reporting use of different food sources

	Overall n (%)	Kisumu n (%)	Homabay n (%)	P-value for difference between study areas**
**Food source (n=357)**				
**Purchased**	356 (100)	176 (100)	180 (99)	1.0
**Own produce**	124 (35)	35 (20)	89 (49)	< 0.001*
**Own livestock**	33 (9)	14 (8)	19 (11)	0.41
**Gift**	17 (5)	12 (7)	5 (3)	0.085
**Relief**	4 (1)	1 (1)	3 (2)	0.62
**Payment in kind**	2 (1)	0	2 (1)	0.50
**Gather/hunt/fish**	5 (1)	0	5 (3)	0.061
**Barter**	0	0	0	-
**Other**	0	0	0	-
**Food retail source (n=357)**				
**Supermarket**	180 (50)	87 (49)	93 (51)	0.71
**Open air market**	339 (95)	161 (91)	178 (98)	0.003*
**Kiosk**	275 (77)	143 (81)	132 (73)	0.062
**General shop**	144 (40)	89 (51)	55 (30)	< 0.001*
**Specialised shop**	46 (13)	44 (25)	2 (1)	< 0.001*
**Informal (roadside) vendor**	74 (21)	44 (25)	30 (17)	0.050
**Restaurant**	16 (4)	8 (4)	8 (4)	0.95
**Fast food**	9 (3)	4 (2)	5 (3)	1.0
**Café**	1 (0)	0	1 (0)	1.0
**Online**	1 (0)	1 (0)	0	0.49
**Other**	0	0	0	-

*significant at the 0.05 level
**Pearson's chi-squared test or Fisher's exact test was used to determine statistical differences between study areas

Supplementary Table S9 Association between household food source and food retail source with severe household food insecurity

	**Outcome:** Severe household food insecurity	
	**Crude odds ratio (95% confidence interval)**	**Adjusted odds ratio (95% confidence interval)**
**Food source (yes/no)**		
Own produce (374/2)	1.31 (0.85, 2.01)	1.07 (0.57, 2.01)
Own livestock (133/243)	**0.36 (0.17, 0.74)***	0.40 (0.16, 1.02)
Gift (17/359)	1.33 (0.48, 3.68)	1.38 (0.39, 4.82)
Relief (4/372)	2.17 (0.22, 21.02)	0.44 (0.04, 5.40)
Payment in kind (2/374)	0.72 (0.04, 11.53)	2.49 (0.00, 1,526.42)
Gather/hunt/fish (5/371)	2.90 (0.32, 26.22)	1.89 (0.12, 30.90)
**Food retail source (yes/no)**		
Supermarket (190/186)	**0.30 (0.19, 0.46)***	0.69 (0.40, 1.18)
Open air market (357/19)	1.02 (0.40, 2.59)	1.15 (0.37, 3.60)
Kiosk (286/90)	0.97 (0.60, 1.56)	0.98 (0.51, 1.87)
General shop (151/225)	0.73 (0.48, 1.11)	0.78 (0.45, 1.36)
Specialised shop (46/330)	**0.41 (0.22, 0.78)***	0.73 (0.31, 1.72)
Informal (roadside) vendor (78/298)	1.19 (0.71, 1.98)	0.79 (0.42, 1.50)
Restaurant (16/360)	**0.22 (0.07, 0.71)***	0.30 (0.08, 1.14)
Fast food (9/367)	0.35 (0.09, 1.42)	0.87 (0.12, 6.21)

Note: “Purchased”, “Barter”, “Other” food sources and “Café”, “Online”, and “Other” food retail sources were omitted due to collinearity.

Odds of moderate or severe household food insecurity in those using the food source vs not using the food source. *significant at the 0.05 level.

Crude model: Unadjusted, n = 376

Adjusted model: Adjusted for study area, household head characteristics (age, sex, highest educational qualification, and working status of household head), household size and years lived in dwelling, asset-based measures of socioeconomic status (type of dwelling, electricity, main water source, owning a fridge, owning/having access to a private car), n = 368 (8 households missing data for household head)

Supplementary Table S10 Association of household food source and food retail source with achieving minimum dietary diversity

	**Outcome:** Achieved minimum dietary diversity	
	**Crude odds ratio (95% confidence interval)**	**Adjusted odds ratio (95% confidence interval)**
**Food source**		
Own produce (124/233)	1.06 (0.70, 1.60)	1.51 (0.93, 2.43)
Own livestock (33/324)	1.53 (0.87, 2.69)	1.81 (0.96, 3.41)
Payment in kind (2/355)	0.62 (0.04, 10.08)	0.45 (0.03, 6.87)
**Food retail source**		
Supermarket (180/177)	**3.07 (2.03, 4.67)***	**2.84 (1.79, 4.49)***
Open air market (339/18)	0.50 (0.20, 1.24)	0.57 (0.22, 1.44)
Kiosk (275/82)	**1.63 (1.00, 2.63)***	**1.67 (1.02, 2.74)***
General shop (144/213)	0.91 (0.61, 1.37)	0.76 (0.49, 1.19)
Specialised shop (46/311)	1.70 (0.94, 3.08)	1.30 (0.67, 2.52)
Informal (roadside) vendor (74/283)	1.60 (0.96, 2.66)	1.50 (0.87, 2.58)
Restaurant (16/341)	3.62 (0.91, 14.42)	3.17 (0.80, 12.64)
Fast food (9/348)	1.78 (0.51, 6.14)	1.68 (0.42, 6.74)

Note: “Purchased”, “Barter”, “Relief”, and “Other” food sources and “Café”, “Online”, and “Other” food retail sources were omitted due to collinearity.

Odds of achieving minimum dietary diversity in those using the food source vs not using the food source. *significant at the 0.05 level

Crude model: Unadjusted, n = 493 (357 clusters)

Adjusted model: adjusted for study area, age and sex, highest educational qualification, working status and household size, non-communicable diseases (hypertension, diabetes, raised blood cholesterol, heart disease, stroke, cancer), self-rated overall health and physical activity, n = 493 (357 clusters)

Supplementary Table S11 Association of household food source and food retail source with household food insecurity

	**Adjusted odds ratio (95% confidence interval)**	
	Moderate or severe household food insecurity	Severe household food insecurity
**Food source (yes/no)**		
Own produce (112/179)	1.31 (0.52, 3.30)	1.21 (0.66, 2.23)
Own livestock (24/267)	**0.25 (0.08, 0.81)***	0.42 (0.16, 1.11)
Gift (13/278)	2.07 (0.24, 17.55)	2.01 (0.48, 8.44)
Relief (3/288)	Omitted	0.66 (0.05, 8.18)
Payment in kind (2/289)	0.20 (0.006, 6.72)	0.80 (0.04, 16.58)
Gather/hunt/fish (5/286)	Omitted	1.70 (0.17, 17.16)
**Food retail source (yes/no)**		
Supermarket (152/139)	**0.16 (0.05, 0.51)***	**0.47 (0.27, 0.83)***
Open air market (280/11)	1.18 (0.13, 11.15)	1.03 (0.23, 4.63)
Kiosk (221/70)	1.61 (0.68, 3.79)	1.17 (0.60, 2.26)
General shop (115/176)	0.93 (0.42, 2.03)	0.87 (0.49, 1.52)
Specialised shop (38/253)	**0.21 (0.07, 0.66)***	0.50 (0.22, 1.18)
Informal (roadside) vendor (54/237)	0.90 (0.33, 2.44)	1.02 (0.52, 2.01)
Restaurant (12/279)	0.27 (0.06, 1.20)	0.30 (0.07, 1.27)
Fast food (7/284)	0.29 (0.04, 2.04)	1.86 (0.21, 16.48)

Note: "Purchased", "Barter", "Relief", and "Other" food sources and "Café", "Online", and "Other" food retail sources were omitted due to collinearity.

Odds of household food insecurity in those using the food source vs not using the food source.
*significant at the 0.05 level

Adjusted for study area, household head characteristics (age, sex, highest educational qualification, and working status of household head), household size, years lived in dwelling, average gross monthly household income per household member, n = 291 due to missing data for income (n=77) and household head (n=8)
